# Modeling the Dynamics of Heroin and Illicit Opioid Use Disorder, Treatment, and Recovery

**DOI:** 10.1007/s11538-022-01002-w

**Published:** 2022-03-02

**Authors:** Sandra Cole, Stephen Wirkus

**Affiliations:** 1 School of Mathematical and Statistical Sciences, Arizona State University, Tempe, AZ USA; 2 School of Mathematical and Natural Sciences, Arizona State University, Glendale, AZ 85306 USA

**Keywords:** Population biology, Backward bifurcation, Mathematical epidemiology, Compartmental model, Heroin use disorder, Drug addiction

## Abstract

Opioid use disorder (OUD) has become a serious leading health issue in the USA leading to addiction, disability, or death by overdose. Research has shown that OUD can lead to a chronic lifelong disorder with greater risk for relapse and accidental overdose deaths. While the prescription opioid epidemic is a relatively new phenomenon, illicit opioid use via heroin has been around for decades. Recently, additional illicit opioids such as fentanyl have become increasingly available and problematic. We propose a mathematical model that focuses on illicit OUD and includes a class for recovered users but allows for individuals to either remain in or relapse back to the illicit OUD class. Therefore, in our model, individuals may cycle in and out of three different classes: illicit OUD, treatment, and recovered. We additionally include a treatment function with saturation, as it has been shown there is limited accessibility to specialty treatment facilities. We used 2002–2019 SAMHSA and CDC data for the US population, scaled to a medium-sized city, to obtain parameter estimates for the specific case of heroin. We found that the overdose death rate has been increasing linearly since around 2011, likely due to the increased presence of fentanyl in the heroin supply. Extrapolation of this overdose death rate, together with the obtained parameter estimates, predict that by 2038 no endemic equilibrium will exist and the only stable equilibrium will correspond to the absence of heroin use disorder in the population. There is a range of parameter values that will give rise to a backward bifurcation above a critical saturation of treatment availability. We show this for a range of overdose death rate values, thus illustrating the critical role played by the availability of specialty treatment facilities. Sensitivity analysis consistently shows the significant role of people entering treatment on their own accord, which suggests the importance of removing two of the most prevalent SAMHSA-determined reasons that individuals do not enter treatment: financial constraints and the stigma of seeking treatment for heroin use disorder.

## Introduction

A national crisis has emerged regarding opioid use disorder (OUD) (Vivolo-Kantor et al. 2018). Opioid overdose rates are on the rise and opioids are the primary cause of overdose deaths in the USA (Vivolo-Kantor et al. 2018; Jalal et al. 2018). In 2009, more than 20,000 people died in the USA by overdosing on opioids, including prescription opioids, heroin, and illicitly manufactured fentanyl; in 2019, the number of yearly opioid overdose deaths increased to nearly 50,000 according to the National Institute on Drug Abuse (Centers for Disease Control and Prevention 2020; National Institutes of Health 2019). Prescription opioid overdose, abuse, and dependence accounts for a total cost of 78.5 billion dollars a year reported by the Centers for Disease Control and Prevention (CDC). These costs are the result of elevated health care, drug abuse treatment, criminal justice, and loss of productivity expenditures (National Institutes of Health 2019; Florence et al. 2016). Other consequences of opioid abuse and dependence are exposure to sexually transmitted diseases, bacterial infections, and Neonatal abstinence syndrome (Hartnett et al. 2019; Centers for Disease Control and Prevention 2017; Haight et al. 2018; Volkow 2018). In addition, drug abuse is being closely linked to major depressive disorders and suicide attempts, which is now one of the increasing causes of death in the USA, according to the CDC (Dragisic et al. 2015; Center for Disease Control and Prevention 2016; Brook et al. 2002; Cole et al. 2019).

Opioids were commonly known in the past as naturally occurring substances derived from the opium poppy plant. They were thought to reduce the suffering of pain safely and effectively. Today, opioids now include the semisynthetic and fully synthetic drugs which invoke more intense, longer-lasting feelings of euphoria. Whether natural or synthetic, once in the bloodstream and traveled to the brain, they bind to $\mu$-opioid receptors. This triggers the same reward system of pleasure and pain relief as do our body’s naturally occurring opioids called endorphins. The opioids activate the mid part of our brain generating feelings of pleasure from the discharge of dopamine in another part of our brain. This is known as the mesolimbic reward system (Kosten and George 2002; Lyden and Binswanger 2019; Incze and Steiger 2019; Veilleux et al. 2010).

Simultaneously, another part of the brain is remembering those good feelings of pleasure, specifically the details surrounding the event. Later, when faced with a similar situation, cravings for the drug taken are encountered. This is termed conditioned associations and it makes it very difficult for the user to not seek out that past feeling of pleasure. This leads to repeated use especially in the early stages. However, over time, repeated use switches from invoking those feelings of pleasure to avoiding the bad feelings of withdrawal. Another consequence of consistent opioid use is tolerance, which occurs when higher doses are needed to create the same previous sought after effects. The brain adjusted and now the individual feels right-minded when opioids are present, but abnormal when they are not, making withdrawal symptoms and cravings an issue. The implication of these complex brain processes then lead to the underlying causes for continuing use where a vicious cycle of repeated drug use has begun (Kosten and George 2002).

In recent years, opioid analgesics have been overprescribed and given their effect on the brain, this has resulted in an increased risk of OUD. This has also influenced an increase of heroin use where multiple users (4 out of 5 reported) have switched over from opioid pain reliever prescriptions because of lower cost and accessibility issues (Kolodny et al. 2015; Volkow 2018; Schuckit 2016; Connery 2015).

Fentanyl and other potent synthetic opioids on the black market have also fueled this problem. They are less expensive, more potent, and less costly to import and are either used to adulterate the heroin or replace it. The adulterated outcome of heroin mixed with fentanyl or other synthetics is unpredictable and dangerous (Volkow 2018; Williams et al. 2017; Spencer et al. 2019; Lyden and Binswanger 2019).

Many articles report a great need for OUD treatment, largely unmet, that signals a serious, widespread public health concern in the USA. For example, only about half of those individuals with heroin use disorder in the USA received treatment as stated in a 2014–15 study. Reasons mentioned include treatment not easily accessible, shortages of trained healthcare staff, insurance coverage issues, limited policy changes, limited financing of care, and limited means of quality care (Ghitza and Tai 2014; Mojtabai et al. 2019; Kolodny et al. 2015; Volkow 2018; Williams et al. 2019; Connery 2015; National Institutes of Health 2021).

Other than the previously mentioned obstacles, another barrier for treatment and limited access to care may include that the public’s view of drug abuse and dependence is stigmatized as opposed to being viewed as a chronic life-threatening disease in need of assistance. As a result of the stigma, in the past, the focus was on an abstinence-based treatment plan. Currently, there are three medications approved by the Food and Drug Administration (FDA) proven to reduce future overdoses and illicit drug use when combined with counseling and behavioral therapies. However, there still exists some reluctance on using these medications to treat OUD. The three medications are methadone, buprenorphine, and extended-release naltrexone. The combination of medication with counseling and behavioral therapies is called medication-assisted treatment (MAT) (Mojtabai et al. 2019; Volkow 2018; Coffa and Snyder 2019; Williams et al. 2019; Lyden and Binswanger 2019; SAMHSA 2021; National Institutes of Health 2021).

These medications remain underused where only a minority receives any treatment (including non-medication routes) and even a smaller amount receive MAT. Among treatment programs in the private sector, less than fifty percent offer opioid based medications and of these programs only thirty-three percent of patients are prescribed them. Therefore, many of the 2.4 million in the USA with OUD do not receive any MAT. To diminish the US OUD overdose epidemic, these barriers and misunderstandings for using these treatment steps must be tackled. OUD treatment is important to decrease the mortality of millions of Americans at risk of opioid-related overdoses. As a result, public health authorities are increasing efforts to integrate such treatment (Mojtabai et al. 2019; Kolodny et al. 2015; Volkow 2018; Williams et al. 2017; Lyden and Binswanger 2019; National Institutes of Health 2021)

A tool that can be used for understanding the complex issues surrounding OUD and illicit OUD, its treatment options, and methods for decreasing relapse, is a mathematical model. Mathematical models are very important to gain understanding of disease epidemiology. Using the spread of OUD viewed as the potential contagion, we can then use a mathematical model to describe the spread of OUD and the dynamics underlying those patterns that can best inform and assist policy makers in targeting prevention and treatment resources for maximum effectiveness (Bailey 1975; Anderson and May 1992; Murray 2007; Brauer and Castillo-Chavez 2012).

Studies of mathematical models on drug use have been previously conducted. White and Comiskey (2007) divided the population into susceptible, current, and in-treatment drug users for heroin addiction. A basic reproductive number, representing how many new users is produced per each current user, was found. A sensitivity analysis pertaining to control efforts was performed, which found that decreasing the transmission term of the contagion showed higher significance than increasing the proportion of users who enter treatment. The authors also found a condition where a backward bifurcation exists, which means that an endemic equilibrium may exist even when the reproductive number is less than one. Therefore, extra efforts would be needed to drive down the epidemic. Also noted in their model is the inclusion of enhanced death rates for the current users and users-in-treatment classes.

Some model studies of the White and Comiskey article were considered by other authors Mulone and Straughan (2009), Wang et al. (2011), Muroya et al. (2014), Ma et al. (2017) including Wangari and Stone.

Wangari and Stone (2017) had the added compartment class of individuals who left treatment but are not using. They also added a saturation term to deal with the shortcomings of the healthcare system when too many people seek treatment at the same time. They found when this saturation parameter was above a critical threshold, backward bifurcation existed. Their sensitivity analysis concluded that this parameter was of high importance in feeding the epidemic. The effective contact rate and relapse rate from treatment are other parameters they found with high sensitivity.

Additional models branched off of the White and Comiskey as well, including the distributed time delay (Liu and Zhang 2011; Liu and Wang 2016; Fang et al. 2014; Huang and Liu 2013; Samanta 2011) and the age structured models (Fang et al. 2015b, a; Wang et al. 2019).

Caldwell et al. (2019) implemented and analyzed a Vicodin epidemic model that focused only on the population of people who were prescribed Vicodin. They also included a global sensitivity analysis to show that preventative measures over treatment efforts are more successful for reduction of misuse.

Battista et al. (2019) proposed a model that added an opioid prescription drug user class where a potential user can become addicted through either the use of prescriptions, legally or illicitly, or through contact with another addicted person; they included a treatment class as well. Mathematical analysis was performed, showing that an addiction-free state cannot be attained without controls over prescriptions. Their sensitivity analysis showed that prevention, followed by vigorous treatment, may result in a low status of endemic misuse.

We propose an “illicit opioid use disorder” (IOUD) model to describe the role that black market opioids such as heroin, fentanyl, and other synthetic opioids play in the current opioid epidemic. Our model does not include a prescription class but will be extended to do so in future work. Thus, our proposed IOUD model can be viewed as what might happen if opioids were outlawed or, perhaps, severely restricted.

Novel to our model is the inclusion of a recovered class that does not allow for a past user to ever be considered as a nonusing susceptible individual in the future. Therefore, we must allow for relapse from both the recovered and treatment classes. According to Kosten and George (2002), repeated and prolonged drug use modifies physiological brain functions. Moreover, alternating between abstinence and withdrawal creates a “changed set point” model. Within this model, healthy dopamine (DA) transmitter activity is permanently altered by use of opioids. This effectively changes the natural baseline of DA tolerance in addicted individuals. Another model called the “cognitive deficits model of drug addiction” explains that damage to the prefrontal cortex may result due to habitual use. This further reduces judgment capacity and impulse constraint. The challenges arising from this neurobiological deterioration permanently increases the risk of relapse. Since chronic opioid use results in these brain transformations, cravings may be produced, causing a recovered individual who is no longer opioid dependent to relapse, following months or years of their abstinence (Kosten and George 2002; Kolodny et al. 2015).

Our IOUD model also considers a treatment class with a saturation term that slows down the rate at which people receive treatment, due to the previously mentioned barriers. We will see that both of these extensions play a role in the dynamics of the system.

## Model Formulation and Basic Properties

**Fig. 1:**
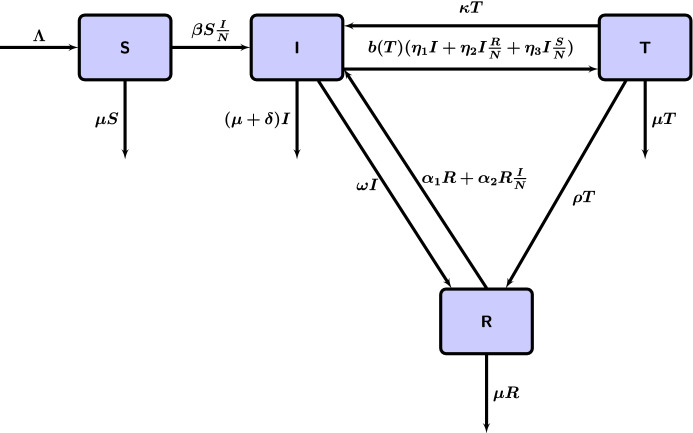
Compartmental flow diagram of the illicit opioid use disorder (IOUD) model. *S* represents susceptible individuals, *I* represents individuals with illicit OUD, *T* represents those in specialty treatment facilities, and *R* represents recovered individuals. *R* is considered distinct from *S* due to an increased potential for relapse. The factor $b(T)=\frac{1}{1+\epsilon T}$ models the decreased rate of entrance into the *T* class due to limited access of care in specialty treatment facilities

Our proposed model assumes a homogeneous mixing of the human population. The total population at time *t* is denoted by *N*(*t*) and is divided into four mutually exclusive compartments as follows: susceptibles *S*(*t*), individuals with illicit OUD *I*(*t*), individuals in a treatment facility *T*(*t*), and recovered individuals *R*(*t*). Thus, $N(t)=S(t)+I(t)+T(t)+R(t)$; see Fig. 1 for how individuals can move between compartments.

$${{\textit{Susceptibles }}(S(t)):}$$

The susceptible (potential individuals with illict OUD) class describes the number of the population who either have never used opioids or have used illicit opioids but never been considered to have illicit OUD. The susceptible population is increased by the constant recruitment rate, $\Lambda$. A constant for recruitment was chosen because it will lead to an asymptotically constant population size as opposed to a linear one which might possibly lead to exponential growth or decay.

$${{\textit{IOUD }}(I(t)):}$$

The IOUD class describes the individuals who have illicit OUD. OUD according to the *Diagnostic and Statistical Manual of Mental Disorders, 5th Ed.* is defined as the use of opioids leading to a precarious situation of repeated use and as a result, at least two destructive symptoms occur within a year period. These include problems such as strong, persistent cravings, failure to perform societal and personal obligations, increased physical endangerment, and an increased tolerance to opioids. A full list can be found in the manual (Edition et al 2013).

Someone who takes opioids illicitly a few times, in some kind of social circumstance (a few parties, music festivals, etc), but never has the kind of constant use that would result in the patterns discussed above would not be considered as having illicit OUD. Thus, this individual would not be considered in the IOUD class but will remain in the susceptible class.

This population class is considered infectious and as a consequence of interacting with individuals with illicit OUD, a susceptible individual may develop tendencies that could lead to illicit OUD. The value $\beta$ is the transmission rate of that interaction resulting in a change of class from *S* to *I*. In this way, the susceptible population may flow to the IOUD class.

There are multiple ways that individuals transition out of the IOUD.

$${{\textit{Treatment class }}(T(t)):}$$

The treatment class describes individuals with illicit OUD who are in a specialty treatment facility. Individuals with illicit OUD may decide to leave for the treatment class on their own at a rate of $\eta _1$, or through the influence of a recovered individual or someone from the susceptible population; the last two interaction rates are $\eta _2$, $\eta _3$, respectively. These individuals may relapse back to the IOUD class by relapse rate $\kappa$ or at the end of their treatment they may flow to the recovered class at rate $\rho$.

From the yearly statistics from the Substance Abuse and Mental Health Services Administration (SAMHSA) published in the annual National Survey on Drug Use and Health (NSDUH), specialty treatment facilities (our *T* class) include hospitals (inpatient only), rehabilitation facilities (inpatient or outpatient), or mental health centers. In contrast, non-specialty treatment facilities include emergency room, private doctor’s office, self-help group, and prison/jail (Center for Behavioral Health Statistics and Quality 2020).

$${{\textit{Recovered }}(R(t)):}$$

The recovered class describes all the individuals who either completed specialty treatment (i.e., went from *T*(*t*) to *R*(*t*)), or those with illicit OUD who quit on their own or with the help of a non-specialty treatment facility (i.e., went from *I*(*t*) to *R*(*t*) in either case).

Since illicit opioid use is a chronic condition (Kosten and George 2002), individuals remain in the recovered state unless they relapse which may be on their own at a rate of $\alpha _1$ or as a consequence of interacting with an individual in the IOUD class at a rate of $\alpha _2$.

There is a removal from each class as the natural death rate $\mu$, whereas the IOUD class, *I*(*t*), has an additional removal rate of $\delta$. With this, the added component due to illicit OUD overdose death (Seth et al. 2018), an overall computed death rate for the individuals with IOUD would be $\mu +\delta$.

### Model Equations

The model is given by the following deterministic system of nonlinear differential equations:

1 $$\begin{aligned} \left\{ \begin{aligned} \frac{\mathrm{d}S(t)}{\mathrm{d}t}&= \Lambda - \beta S\frac{I}{N} - \mu S, \\ \frac{\mathrm{d}I(t)}{\mathrm{d}t}&= \beta S\frac{I}{N}+\alpha _1 R + \alpha _2 R\frac{I}{N}+\kappa T\\&\quad -b(T)\left( \eta _1 I+\eta _2\frac{R}{N}I+\eta _3\frac{S}{N} I\right) -(\omega +\mu +\delta )I,\\ \frac{\mathrm{d}T(t)}{\mathrm{d}t}&= b(T)\left( \eta _1I+\eta _2\frac{R}{N}I+\eta _3\frac{S}{N} I\right) -(\kappa +\rho +\mu )T,\\ \frac{\mathrm{d}R(t)}{\mathrm{d}t}&= \omega I +\rho T -(\alpha _1 +\mu )R-\alpha _2R\frac{I}{N}. \end{aligned} \right. \end{aligned}$$

where $b(T)=\frac{1}{1+\epsilon T}$ and all parameters are nonnegative.

We use a saturation treatment function *b*(*T*) to modify the flow of individuals with illicit OUD to treatment, where the parameter $\epsilon$ models a saturation of availability of specialty treatment facilities. This limits the amount of individuals with illicit OUD that can go into specialty treatment facilities due to the limited access of care discussed previously in the introduction.

A description of variable and parameter values are listed in Table 1.

**Table 1:** Description of variables and parameters of the model

Variable	Description
*S*(*t*)	The total number of people who are susceptible at time *t*
*I*(*t*)	The total number of individuals with illicit OUD (for the first time and from relapse) not in specialty treatment or recovered at time *t*
*T*(*t*)	The total number of individuals in specialty treatment at time *t*
*R*(*t*)	The total number of individuals who have either completed specialty or non-specialty treatment or “quit cold turkey” at time *t*

Parameter	Description
*N*	Size of the total population
$\Lambda$	The rate of the number of individuals entering the susceptible population
$\mu$	The natural death rate of the general population
$\beta$	The transmission rate of becoming an individual with illicit OUD through interaction with others in the IOUD class
$\eta _1$	The rate of individuals in *I* who enter specialty treatment on their own
$\eta _2$	The rate of individuals in *I* who enter specialty treatment through interaction with a recovered individual
$\eta _3$	The rate of individuals in *I* who enter specialty treatment through interaction with a susceptible individual
$\omega$	The rate of individuals in *I* who enter the recovered class by either completing treatment in non-specialty facilities and/or “quitting cold turkey”
$\rho$	The rate of individuals leaving treatment and entering the recovered class
$\kappa$	The rate of individuals leaving treatment and returning to the *I* class
$\alpha _1$	The rate of individuals in the recovered state relapsing back to the *I* class on their own
$\alpha _2$	The rate of individuals in the recovered state relapsing back to the *I* class through interaction with an individual in the *I* class
$\delta$	Death rate of individuals in the *I* class due to overdose
$\epsilon$	Saturation term for entering a specialty treatment facility

The basic properties of the IOUD model were explored and those results can be found in Appendix.

**Fig. 2:**
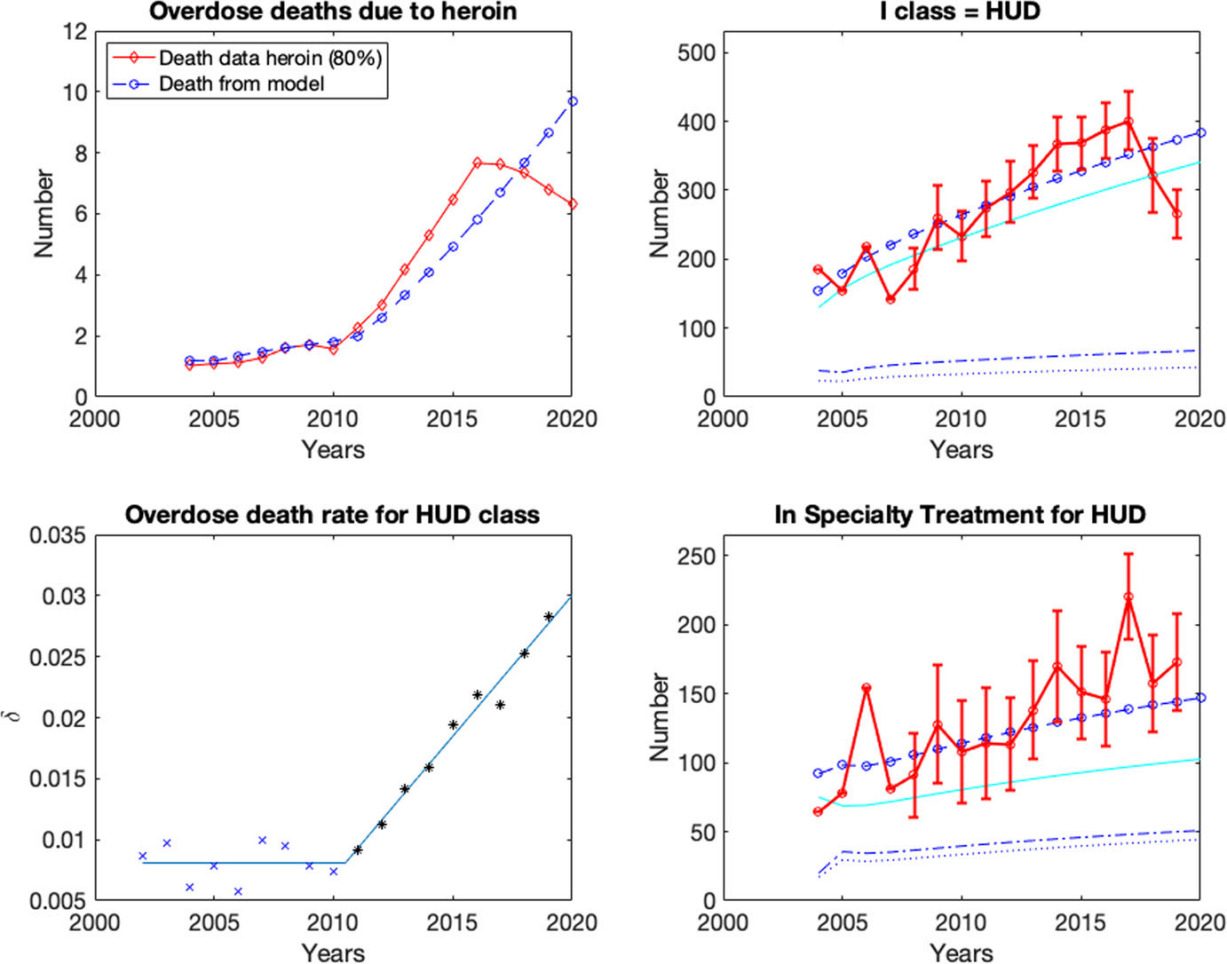
Model output compared to data scaled to a population of 200,000 by taking into account the yearly US population values. (Top Left): CDC data for overdose deaths in HUD class due to heroin, obtained as 0.8$\times$ (total overdose deaths due to heroin), presented as red curve with diamonds compared with model output as blue curve with circles. (Top right): SAMHSA data for in “HUD in past year,” with error bars when given. Model approximation is the blue curve with circles, calculated with instantaneous model variable *I* (solid, cyan curve immediately below) averaged over each year and added to the “correction” for those that left and also possibly returned to *I* (see text). (Bottom right): SAMHSA data for in “specialty treatment in past year coming from *I*,” with error bars when given. Model approximation is the blue curve with circles, calculated with instantaneous model variable *T* (solid, cyan curve immediately below) averaged over each year and added to the “correction” for those that left and also possibly returned to *T* (see text). The bottom 2 curves in the right panels signify those who left *I* and *T* over the year presented with dash–dot curves and the corrected quantities of those who left the respective classes are presented with dotted curves. These last two quantities sum to give the solid curve with circles that we compare with the SAMHSA data. (Bottom left): Data-derived and least squares fit for $\delta$. Asterisks and x-marks are calculated from data (see text and equation (3)) with blue x-marks used to obtain the horizontal (constant) line and black asterisks used to obtain the nonzero sloped line; both lines are calculated with a least squares fit (Color figure online)

## Data Explanation and Parameter Estimation

Our model considers illicit OUD, treatment, and recovery, as well as overdose deaths. CDC data exists for overdose deaths due to synthetic opioids (primarily fentanyl) as well as heroin (sometimes in combination with synthetic opioids). However, the data from SAMHSA on illicit OUD and treatment is limited to heroin likely because the presence of synthetic opioids is a relatively recent phenomenon. Thus, for the purpose of comparing our model to data, we consider only heroin use or heroin use with synthetic opioids (both considered by SAMHSA) but do not include additional synthetic-opioid-only use. We consider a generic US city of approximately 200,000 people and scale the national data from the number of individuals with heroin use disorder (HUD), the number of individuals with treatment, and the number of overdose deaths to a city of this size by taking into account the increasing yearly US population. This allows us to consider a nearly constant population size as we analyze the dynamics of the model. For example, heroin use disorder (HUD) in 2002 in the top right graph of Fig. 2 is 148.97 = (214,000/287.3E+06) $\times$ 200,000; see Table 2 for similar yearly numbers. We found CDC data with the number of deaths due to heroin or heroin mixed with synthetic opioids (third column of Table 2), which is dominated by fentanyl, as well as death from synthetic opioids alone (second column). SAMHSA data was found for the number of individuals with HUD, with the NSDUH counting those with HUD in the past year (fifth column, relates to our variable *I*). SAMHSA data is available for treatment in a specialty facility in the past year (sixth column, relates to our variable *T*) and also in a general treatment center in the past year (not presented). Data for "in specialty treatment for HUD" was only presented in the 2014–2017 SAMHSA survey results. In order to scale the other years to give an approximate number in specialty treatment facilities coming from HUD (our variable *T*), we looked at the ratio of specialty treatment from HUD to the specialty treatment data in the 4 years when it was available. The factor 0.6874 is the average of the ratio. The specialty treatment  $\times$0.6874 is labeled in the sixth column with an asterisk, and also given without error bars in the graph. The treatment data, similar to the HUD data, counted individuals in a specialty facility in the last year. This is the data presented in our graphs with the raw data given in Table 2. The error bars in the graphs represent the standard error given in the SAMHSA data (not presented in the table).

**Table 2:** Data for USA, 2002–2020. The number of overdose deaths for 2002–2020 are from the CDC (Centers for Disease Control and Prevention 2020). US population comes from United Nations (2019). Use disorder and specialty treatment data come from SAMHSA’s NSDUH (Center for Behavioral Health Statistics and Quality 2020, 2018, 2016, 2015, 2014; Lipari and Hughes 2015; Center for Behavioral Health Statistics and Quality 2013; Substance Abuse and Mental Health Services Administration 2011, 2010, 2008, 2006). The derivation of values in the column $\delta$-data are given in (3) where we used (HUD class data in year)$\times$0.903 to estimate average number with HUD during the year. The values in the column $\delta$-fit are obtained from (4)

Year	Deaths due to overdose		US population	HUD in last year	Specialty treatment in last year from *I*	$\delta$-data	$\delta$-fit
	Synthetics	Heroin					
2002	1295	2089	287.3E+06	214,000	Not available	0.008648	0.008089
2003	1400	2080	289.8E+06	189,000	Not available	0.009750	0.008089
2004	1664	1878	292.4E+06	270,000	107,200$^*$	0.006162	0.008089
2005	1742	2009	295.0E+06	227,000	130,600$^*$	0.007841	0.008089
2006	2707	2088	297.8E+06	324,000	259,100$^*$	0.005709	0.008089
2007	2213	2399	300.6E+06	214,000	138,200$^*$	0.009932	0.008089
2008	2306	3041	303.5E+06	283,000	156,000$^*$	0.009520	0.008089
2009	2946	3278	306.3E+06	369,000	221,300$^*$	0.007870	0.008089
2010	3007	3036	309.0E+06	361,000	188,300$^*$	0.007451	0.008089
2011	2666	4397	311.6E+06	426,000	200,700$^*$	0.009144	0.009255
2012	2628	5925	314.0E+06	467,000	201,400$^*$	0.011240	0.01156
2013	3105	8257	316.4E+06	517,000	246,800$^*$	0.014149	0.01387
2014	5544	10,574	318.7E+06	586,000	270,000	0.015986	0.01618
2015	9580	12,989	320.9E+06	591,000	242,000	0.019471	0.01848
2016	19,413	15,469	323.0E+06	626,000	235,000	0.021892	0.02079
2017	28,466	15,482	325.1E+06	652,000	358,000	0.021037	0.02310
2018	31,335	14,996	327.1E+06	526,000	291,500$^*$	0.025258	0.02540
2019	36,259	14,019	329.1 E+06	438,000	321,000$^*$	0.028356	0.02771
2020	56,883	13,058	331.0E+06	Not available	Not available	Not available	0.03002

$^{*}$Specialty treatment$\times$0.6874 because specialty treatment from HUD only asked in 2014–2017 SAMHSA surveys. The factor 0.6874 is the average of the ratio of specialty treatment from HUD to specialty treatment in the 4 years when data is available

Our variables *I* and *T* are instantaneous in time, whereas the SAMHSA data gives those in the respective classes *in the past year*. In the case of comparing our model output with the SAMHSA treatment data, individuals that were in treatment in the past year could (i) be currently in *T*, (ii) have relapsed and went from *T* back to *I*, or (iii) have successfully completed treatment and moved from *T* to *R* in the past year. In the case of comparing our model output with the SAMHSA HUD data, individuals with HUD could (i) be currently in *I*, (ii) have moved to treatment (*I* to *T*), or (iii) moved directly from *I* to *R* in the past year. Thus, we additionally need to keep track of the number of individuals who left each of these classes each year. We further correct our model output with a small discount for those that went back again (after having left and thus should not have been discounted). In the data matching plot, we present the model output, those that left *I* and *T* over the year, and a correction of those who left *I* and *T* over the year but then went back (estimated with $\kappa T_{\small {I}}+\alpha _1R_{\small {I}}$, and $I_{\small {T}}\left( \eta _1+\eta _3\frac{S}{N} \right) /(1+\epsilon T)$, respectively). Those who left *I* and *T* over the year are presented with dash–dot curves, the corrected quantities of those who left the respective classes are presented with dotted curves, and the variable output of the class is a solid curve with no circles. These last two quantities sum to give the solid curve with circles that we compare with the SAMHSA data.

We were able to come up with reasonable estimates for many of the parameters based on the literature. We used $\mu$ from Wangari (2017), Wangari and Stone (2017) where it was assumed that the average person’s lifespan is 80 years old and thus $\mu =1/80$. From Battista et al. (2019), Battista et al. (2019), we obtained an approximate range of $\rho$ as 0.1 to 0.4 Weiss and Rao (2017). We estimated $\kappa$ to be in the range 0.4 to 0.9 from Smyth et al. (2010), Bailey et al. (2013), Weiss and Rao (2017). We set $\Lambda = 2500$ so that the population in the heroin-free model reaches 200,000 for the assumed $\mu$ and for $\delta =0$. For parameters $\eta _1$, $\eta _2$, and $\eta _3$, the entry to treatment rates, we used the range $0.2-0.95$ from Battista et al. (2019) and Wangari and Stone (2017), Zhang and Liu (2008). Both models had only a linear term from their addicted class to treatment, whereas our model has one linear term and two nonlinear terms between the comparable classes. Considering $\eta =\eta _1+\eta _2(R/N)+\eta _3(S/N)$, we set estimates for $\eta _1 = .5$, $\eta _2 = .1$, and $\eta _3=.17$. Similarly, we found a rate from recovery back to HUD from the literature in a study by Gossop et al. (1989). We estimated $\alpha$ to be in the range 0.1 to 1/3 with $\alpha = \alpha _1+\alpha _2\cdot I/N$ and $\alpha _1$ significantly bigger than $\alpha _2$. We used $\alpha _1=.2$, $\alpha _2=.01$. The parameter $\omega$ for going directly from *I* to *R*, either “quitting cold turkey” or quitting through a general (non-specialty) treatment facility, was estimated to be in the range .01 to 0.2 (Wangari and Stone 2017).

The parameters $\beta$ and $\epsilon$ were difficult to determine, so we did parameter estimation with them as well as for $\rho$ and $\omega$ (where we used the above range from the literature for the latter two) (Banks et al. 2013; Banks and Bihari 2001; Cintrón-Arias et al. 2009). While we were able to approximately match the data for *I* and *T*, we were not able to come close to matching the overdose death data for a fixed $\delta$, which increased significantly from 2010 through 2016, even allowing for a possible change in parameters in 2010 (see derivation below and Table 2).

We now consider $\delta$ in more detail. By definition, we have that

2 $$\begin{aligned} \delta = \frac{\text{ HUD } \text{ overdose } \text{ deaths } \text{ due } \text{ to } \text{ heroin } \text{ per } \text{ year }}{\text{ average } \text{ number } \text{ of } \text{ individuals } \text{ with } \text{ HUD } \text{ during } \text{ the } \text{ year }}. \end{aligned}$$

For the numerator, the CDC data gives total yearly overdose deaths due to heroin, irrespective of whether an individual was with HUD or not (Centers for Disease Control and Prevention 2017). We note that the paper by Battista et al. on prescription opioids estimates a discount factor from the literature on what portion of opioid deaths were from someone addicted to opioids to address this analogous problem (Battista et al. 2019). In our current discussion that focuses on HUD, we did not find any comparable statement in the literature regarding the percentage of individuals who die from an overdose of heroin that were in the HUD class (in contrast to those who die from a heroin overdose but are “casual users”). We estimate that 80% of the heroin overdose deaths are from individuals with HUD as a first approximation that can be corrected if data becomes available. For the denominator, we need to estimate the average number of individuals with HUD during the year for a given year since the SAMHSA data gives the cumulative number of those with HUD in the past year (Center for Behavioral Health Statistics and Quality 2014, 2015, 2016, 2018, 2020; Lipari and Hughes 2015; Center for Behavioral Health Statistics and Quality 2013; Substance Abuse and Mental Health Services Administration 2006, 2008, 2010, 2011). In comparing the model output variable *I* with the model calculation to give the number in *I* in the past year (both with results from the parameter estimation), we observed the graphs were shaped similarly (solid cyan curve immediately underneath solid blue curve with circles in the top right graph of Fig. 2). We thus calculated the ratio of the average of the model output *I* over the past year to the model output *I* in the past year (described above) for each year and found its average value to be 0.903. In our calculation of $\delta$, we thus estimated the average number in the HUD class over the year as the SAMHSA data for those individuals with HUD in the past year multiplied by 0.903. Thus, we calculate the yearly $\delta$ values as

3 $$\begin{aligned} \delta =\frac{ (\text{ total } \text{ overdose } \text{ deaths } \text{ due } \text{ to } \text{ heroin } \text{ per } \text{ year}) \cdot \displaystyle \left( \frac{\text{0.8 } \text{ HUD } \text{ overdose } \text{ deaths } \text{ due } \text{ to } \text{ heroin }}{\text{1 } \text{ overdose } \text{ death } \text{ due } \text{ to } \text{ heroin }}\right) }{(\text{ number } \text{ in } \text{ the } \text{ HUD } \text{ class } \text{ in } \text{ past } \text{ year})\cdot (\text{0.903 })}.\nonumber \\ \end{aligned}$$

In examining the data-derived yearly values of $\delta$, we observed a significant year over year increase starting in 2012 through 2019; see the bottom left subgraph in Fig. 2 and Table 2. The 2020 overdose deaths were published recently by the CDC. During the revision of this manuscript, SAMHSA published the 2020 data for HUD; however, they changed the criteria for classifying an individual as HUD, thus making the 2020 data that were released not obviously compatible with the data from 2019 and earlier. Thus, we are not able to include the 2020 $\delta$ value in our parameter estimates. When plotted versus time, the $\delta$-values follow a piecewise linear function (2002–2019), as shown in the bottom left subgraph in Fig. 2. We use the corresponding piecewise function obtained with a least squares fit (last column of Table 2) in our model calculations:

4 $$\begin{aligned} \delta (t) = \left\{ \begin{array}{cc} 0.0080891345, &{} 2002 \le t<2010.4947542468 \\ .0023071201997~t - 4.63036392433, &{} 2010.4947542468\le t <2020,\\ \end{array}\right. \nonumber \\ \end{aligned}$$

where we present this number of significant digits to have agreement to six significant digits when the function switches branches. (In our computations, additional digits are kept.) Incorporating this piecewise function for $\delta$ into our parameter estimation, our baseline values are

5 $$\begin{aligned} \left. \beta =.09, \rho =.1, \epsilon =.0313, \omega =.04~~\right\}&\text{ via } \text{ parameter } \text{ estimation, } \nonumber \\ \left. \begin{array}{r} \alpha _1=.2,\alpha _2=.01,\kappa =.4,\mu =.0125,\\ \eta _1=.5, \eta _2=.1, \eta _3=.17 \end{array} \right\}&\text{ via } \text{ estimation } \text{ from } \text{ the } \text{ literature, } \nonumber \\ \Lambda =2500~~\}&\text{ for } \text{ a } \text{ city } \text{ of }\approx 200,000.\nonumber \\ \end{aligned}$$

We choose our initial conditions to approximately match the scaled data from $t_0=2002$: $S_0=199{,}500, I_0=102, T_0=95, R_0=100$. While our model is for illicit opioid use and not just heroin, only heroin data is available for the *I* and *T* classes, and that is the data we use to fit our model. The data match is provided in Fig. 2.

Given the myriad of ways in which we varied parameters to try to match the data, we conclude that we cannot fix $\delta$ at a constant but must vary it according to the yearly data if we are to obtain agreement of model output with data. This increase in $\delta$ over time corresponds to a higher overdose death rate per individual in the HUD class. Given the agreement of data and model output, necessitated by an increasing $\delta$, we interpret this deadlier $\delta$ as follows: *the increase in number of heroin overdose deaths was driven by the increase in the prevalence of fentanyl in the heroin supply, as no increase in the HUD class (either seen in the SAMHSA data or our model output) could account for such a drastic increase with a fixed* $\delta$. Fentanyl first appeared in 2007, is 100 times more potent than heroin, and its prevalence is well known to have been getting greater in the heroin supply and illicit opioid use (Worth and House 2018; United States Drug Enforcement Administration 2021).

We note that the number of overdose deaths and the number in the HUD class both have decreased over the last 3 years, whereas our model continues to increase. (Interestingly, the data-derived $\delta$-value for 2018, 2019 still increases in spite of this decrease.) As shown in the second column of Table 2, deaths due to synthetic opioids have skyrocketed. Numerous articles suggest that heroin users may be switching to synthetic opioids, but SAMHSA data does not keep track of synthetic opioid use explicitly and only in the last few years has considered illicit opioid use. A recent article from the RAND corporation states that “Cheap, accessible, and mass-produced synthetic opioids could very well displace heroin, generating important and hard-to-predict consequences” (Pardo et al. 2019).

## Steady-State Analysis

Traditional epidemiological language uses the phrase “disease-free equilibria (DFE)” to describe the absence of the given disease. Our *I* class consists of those active users with illicit OUD. Thus, we will consider an “illicit opioid use disorder-free equilibria” (IOUDFE) that we will shorten to “disorder-free equilibria” (DFE) for convenience. We are interested in the DFE and its stability for (1). With $I,T,R=0$, $\frac{\mathrm{d}S}{\mathrm{d}t}=0$ gives $S^*=\Lambda /\mu$. Hence, the DFE of our IOUD model is $(S^*,I^*,T^*,R^*)= (\Lambda /\mu ,0,0,0)$

For the ensuing analysis, we consider a fixed $\delta$ so that the death rate due to overdose remains constant at some level (e.g., at its 2020 value). We tried to analyze the equilibria of the system using the local stability analysis and the Routh–Hurwitz criterion (Wirkus et al. 2017; Edelstein-Keshet 2005), but due to the complexity of the expressions we were not able to obtain any useful information.

### Calculating the Basic Reproductive Number ${\mathcal {R}}_0$

The basic reproductive number, ${\mathcal {R}}_0$, is a quantity that represents the expected number of new infections produced per infected individual during their infectious period when a disease is introduced into a susceptible population. In the context of our model, it determines the additional number of new individuals with illicit OUD that each individual with illicit OUD will produce before entering treatment or recovery.

We will find the ${\mathcal {R}}_0$ for our model (1) by using the next generation method as presented in Van den Driessche and Watmough (2002) and also by considering a heuristic derivation (see, e.g., Van den Driessche and Watmough 2002; Wangari and Stone 2017); both agree.

We restate (1) with the *b*(*T*) saturation term explicitly in the equations:

6 $$\begin{aligned} \begin{aligned} \frac{\mathrm{{d}}S}{\mathrm{{d}}t}&= \Lambda - \beta S\frac{I}{N} -\mu S, \\ \frac{\mathrm{{d}}I}{\mathrm{{d}}t}&= \beta S\frac{I}{N}+\alpha _1 R + \alpha _2 R\frac{I}{N}+\kappa T - \frac{\eta _1I+\eta _2\frac{IR}{N}+\eta _3\frac{IS}{N}}{1+\epsilon T}-(\omega +\mu +\delta )I,\\ \frac{\mathrm{{d}}T}{\mathrm{{d}}t}&= \frac{\eta _1I+\eta _2\frac{R}{N}I+\eta _3\frac{S}{N} I}{1+\epsilon T} -(\kappa +\rho +\mu )T,\\ \frac{\mathrm{{d}}R}{\mathrm{{d}}t}&= \omega I +\rho T -(\alpha _1 +\mu )R-\alpha _2R\frac{I}{N}. \end{aligned} \end{aligned}$$

For the heuristic derivation of ${\mathcal {R}}_0$, as presented by Van den Driessche and Watmough (2002) we observe that we can cycle in and out of the IOUD class, either through treatment or recovery or both due to relapse of individuals in the treatment or recovery classes (Van den Driessche and Watmough 2002; Wangari and Stone 2017). The average time an individual spends time as an opioid user in *I* without treatment is $U_0=\frac{1}{\mu +\delta +\eta _1+\eta _3+\omega }.$

The fraction of surviving *I* and moving to treatment is $U_1=\frac{\eta _1+\eta _3}{\mu +\delta +\eta _1+\eta _3+\omega }$ and the fraction of surviving *I* and moving to recovered is $W_1=\frac{\omega }{\mu +\delta +\eta _1+\eta _3+\omega }.$ Now, the fraction of the surviving opioid users in *T* returning to *I* is seen to be $U_2=\frac{\kappa }{\mu +\kappa +\rho }$, while the fraction of the surviving opioid users in *R* returning to *I* directly is $W_2=\frac{\alpha _1}{\mu +\alpha _1}.$ We set $r_1=U_1U_2$, which defines going from IOUD to treatment and back to IOUD; and $r_2=W_1W_2$, which defines going from the IOUD class to recovered and back to the IOUD class.

In addition, we now have the fraction of surviving opioid users moving to treatment, then to *R*, and then to *I*:

$$\begin{aligned} r_3=U_1\left( \frac{\rho }{\kappa +\mu +\rho }\right) W_2 \end{aligned}$$

Our new expression for all possible combinations of multiple passes will now be

$$\begin{aligned} 1+(r_1+r_2+r_3) + (r_1+r_2+r_3)^2+(r_1+r_2+r_3)^3+\ldots . \end{aligned}$$

As this is a geometric sequence, we can write its sum as $\frac{1}{1-(r_1+r_2+r_3)}$. Substitution for $r_1$, $r_2$, $r_3$, and multiplication by $\beta U_0$ gives us

7 $$\begin{aligned} {\mathcal {R}}_0= & {} \left( \frac{\beta }{\mu +\delta +\eta _1+\eta _3+\omega }\right) \nonumber \\&\left( \frac{1}{ 1-\frac{\eta _1+\eta _3}{\mu +\delta +\eta _1+\eta _3+\omega }\frac{\kappa }{\mu +\kappa +\rho } -\frac{\omega }{\mu +\delta +\eta _1+\eta _3+\omega }\frac{\alpha _1}{\mu +\alpha _1} -\frac{\eta _1+\eta _3}{\mu +\delta +\eta _1+\eta _3+\omega }\frac{\rho }{\kappa +\mu +\rho } \frac{\alpha _1}{\mu +\alpha _1}}\right) ,\nonumber \\ \end{aligned}$$

which can also be rearranged as

8 $$\begin{aligned} {\mathcal {R}}_0= \frac{\beta (\kappa + \rho + \mu )(\alpha _1 + \mu )}{\left( \begin{array}{l}\alpha _1\delta \kappa + \alpha _1\delta \mu + \alpha _1\delta \rho + \alpha _1\eta _1\mu + \alpha _1\eta _3\mu + \alpha _1\kappa \mu + \alpha _1\mu ^2 + \alpha _1\mu \rho + \delta \kappa \mu + \delta \mu ^2 + \delta \mu \rho \nonumber \\ ~\quad + \eta _1\mu ^2 + \eta _1\mu \rho + \eta _3\mu ^2 + \eta _3\mu \rho + \kappa \mu ^2 + \kappa \mu \omega + \mu ^3 + \mu ^2\omega + \mu ^2\rho + \mu \omega \rho \end{array}\right) }.\\ \end{aligned}$$

In this latter form, we see that entering treatment, either of ones own accord, $\eta _1$, or through the interaction with a susceptible individual, $\eta _3$, as well as recovering on one’s own, $\omega$, (all increasing) will result in a lower value of ${\mathcal {R}}_0$. Decreasing the transmission rate, $\beta$, or increasing the illicit OUD overdose death rate, $\delta$, would also result in decreasing ${\mathcal {R}}_0$. The cycling that can occur between the *I*, *R*, and *T* classes makes the remaining parameters less obvious.

This expression for ${\mathcal {R}}_0$ is the same as that obtained via the next generation method $FV^{-1}$ as we now show (Van den Driessche and Watmough 2002). The $FV^{-1}$ method requires that we identify “new infections” and “infected” compartments. We note that changes of the individual from *T* to *I* and *R* to *I* are not considered to be new infections, but rather the movement of an infected individual through the different compartments. According to the definitions of ${\mathcal {F}}$ and ${\mathcal {V}}$, we compute

$$\begin{aligned} {\mathcal {F}} = \left[ \begin{array}{c} \frac{\beta SI}{N}\\ 0\\ 0\\ 0\\ \end{array}\right] \end{aligned}$$

and

$$\begin{aligned} {\mathcal {V}} = \left[ \begin{array}{c} -\alpha _1 R-\kappa T -\frac{\alpha _2 RI}{N} + \frac{\eta _1 I+\frac{\eta _2 IR}{N}+ \frac{\eta _3 IS}{N} }{\epsilon T+1}+(\omega +\mu +\delta )I\\ \frac{\alpha _2 RI}{N}-\omega I-\rho T+(\alpha _1+\mu )R \\ (\kappa +\rho +\mu )T-\frac{\eta _1 I+\frac{\eta _2 IR}{N}+ \frac{\eta _3 IS}{N} }{\epsilon T+1}\\ -\Lambda + \beta S\frac{I}{N} +\mu S\\ \end{array}\right] \end{aligned}$$

Clearly *I* is an infected compartment as it holds those individuals with IOUD. Due to the structure of the equations and the mathematical method, *T* and *R* must also be considered as infected compartments because individuals can go from *R* or *T* into *I* without interaction because of the non-contact rates between them and the *I* class. In terms of the biological justification, *T* and *R* are infected compartments since opioid use may result in brain transformation with cravings that may be invoked, leading to relapse of an individual in treatment or a recovered individual (Kosten and George 2002). Thus, our infected compartments are *I*, *T*, and *R* giving $m=3$.

According to the definitions of *F* and *V* and using our previously calculated DFE, we obtain

$$\begin{aligned} F = \left[ \begin{array}{ccc} \frac{\beta \Lambda }{\mu N} &{} \quad 0 &{} \quad 0 \\ 0&{}\quad 0&{}\quad 0\\ 0&{}\quad 0&{}\quad 0\\ \end{array}\right] \end{aligned}$$

and

$$\begin{aligned} V = \left[ \begin{matrix} \eta _1+\frac{\eta _3 \Lambda }{\mu N}+\omega +\mu +\delta &{} \quad -\alpha _1 &{} \quad -\kappa \\ -\omega &{} \quad \alpha _1+\mu &{} \quad -\rho \\ -\eta _1-\frac{\eta _3 \Lambda }{\mu N} &{}\quad 0 &{}\quad \kappa + \rho +\mu \\ \end{matrix}\right] . \end{aligned}$$

The calculation of $FV^{-1}$ results in only one nonzero eigenvalue that contains only nonnegative parameter values. This maximum eigenvalue of $FV^{-1}$ gives us the same expression for ${\mathcal {R}}_0$ as from (8).

One interesting observation is the absence of $\epsilon$, $\alpha _2$, and $\eta _2$ from the ${\mathcal {R}}_0$ expression. Since the interpretation of ${\mathcal {R}}_0$ is often stated as one infected introduced into an entirely susceptible population, this would suggest that limited access to special facilities (modeled via $\epsilon$) will not play a role initially and the size of $\frac{RI}{N}$ will be too small for $\alpha _2$ or $\eta _2$ to have any effect.

### Endemic Equilibria

We will now determine the existence of non-trivial endemic equilibria of the system. We will be particularly interested in the situation of a backward bifurcation, which is characterized by a stable endemic equilibria existing even when ${\mathcal {R}}_0<1$. In the region of bi-stability, both the endemic equilibria (EE) and the DFE exist and are stable. We begin by considering the case of no saturation, $\epsilon =0$, so that the situation of limited availability in specialty treatment facilities does not occur:

9 $$\begin{aligned} \left\{ \begin{aligned} \frac{\mathrm{{d}}S}{\mathrm{{d}}t}&= \Lambda - \beta S\frac{I}{N} -\mu S, \\ \frac{\mathrm{{d}}I}{\mathrm{{d}}t}&= \beta S\frac{I}{N}+\alpha _1 R + \alpha _2 R\frac{I}{N}+\kappa T - \left( \eta _1I+\eta _2\frac{IR}{N}+\eta _3\frac{IS}{N}\right) -(\omega +\mu +\delta )I,\\ \frac{\mathrm{{d}}T}{\mathrm{{d}}t}&= \left( \eta _1I+\eta _2\frac{R}{N}I+\eta _3\frac{S}{N} I\right) -(\kappa +\rho +\mu )T,\\ \frac{\mathrm{{d}}R}{\mathrm{{d}}t}&= \omega I +\rho T -(\alpha _1 +\mu )R-\alpha _2R\frac{I}{N}. \end{aligned} \right. \nonumber \\ \end{aligned}$$

We will show that this case does not permit the existence of a backward bifurcation for $\alpha _2=0$ but does permit one for large enough $\alpha _2$. We can obtain an equation in only the variable $I^*$ as follows. We set $\frac{\mathrm{{d}}S}{\mathrm{{d}}t}=0$ and solve for $S^*$:

$$\begin{aligned} S^*=\frac{\Lambda N^*}{I^*\beta +\mu N^*}. \end{aligned}$$

We set $\frac{\mathrm{{d}}N}{\mathrm{{d}}t}=0$ and solve for $N^*$; see (17):

$$\begin{aligned} N^*=\frac{\Lambda -I^*\delta }{\mu }. \end{aligned}$$

We set $\frac{\mathrm{{d}}T}{\mathrm{{d}}t}=0$ and $\frac{\mathrm{{d}}R}{\mathrm{{d}}t}=0$ and solve for $T^*$ and $R^*$:

$$\begin{aligned} T^*= & {} \frac{I\varvec{^*}(I\varvec{^*}N\varvec{^*}\alpha _2 \eta _1 + I\varvec{^*}N\varvec{^*}\eta _2\omega + I\varvec{^*}S\varvec{^*}\alpha _2\eta _3 + (N\varvec{^*})^2\alpha _1\eta _1 + (N\varvec{^*})^2 \eta _1\mu + N\varvec{^*}S\varvec{^*}\alpha _1\eta _3 + N\varvec{^*}S\varvec{^*}\eta _3\mu )}{(I\varvec{^*} \alpha _2\kappa + I\varvec{^*}\alpha _2\mu + I\varvec{^*} \alpha _2\rho - I\varvec{^*}\eta _2\rho + N\varvec{^*} \alpha _1\kappa + N\varvec{^*}\alpha _1\mu + N\varvec{^*} \alpha _1\rho + N\varvec{^*}\kappa \mu + N\varvec{^*}\mu ^2 + N\varvec{^*}\mu \rho )N\varvec{^*}},\\ R^*= & {} \frac{I\varvec{^*}(N\varvec{^*}\eta _1\rho + N\varvec{^*}\kappa \omega + N\varvec{^*}\mu \omega + N\varvec{^*}\omega \rho + S\varvec{^*}\eta _3\rho )}{I\varvec{^*}\alpha _2\kappa + I\varvec{^*}\alpha _2\mu + I\varvec{^*}\alpha _2\rho - I\varvec{^*}\eta _2\rho + N\varvec{^*}\alpha _1\kappa + N\varvec{^*}\alpha _1\mu + N\varvec{^*}\alpha _1\rho + N\varvec{^*}\kappa \mu + N\varvec{^*}\mu ^2 + N\varvec{^*}\mu \rho }, \end{aligned}$$

where $S^*$ and $N^*$ are defined as above.

We substitute $S^*,T^*,R^*$ into $\frac{\mathrm{{d}}I}{\mathrm{{d}}t}=0$. After simplification, we obtain an equation of the form $0=B/C,$ where *B* is a complicated expression involving the parameters as well as $I^*$ and $N^*$ and

$$\begin{aligned} C= & {} ((I^*\beta + N^*\mu )(N^*\mu ^2 + ((\kappa + \alpha _1 + \rho )N^* + I^*\alpha _2)\mu + \alpha _1(\kappa + \rho )N^*\\&\quad + I^*((\alpha _2 - \eta _2)\rho + \kappa \alpha _2))). \end{aligned}$$

We observe from (8) that ${\mathcal {R}}_0$ does not depend on $\eta _2$ or $\alpha _2$ since those factors are not present in ${\mathcal {R}}_0$. Thus, the presence of the factor ($\alpha _2-\eta _2)$ suggests that altering $\eta _2$ or $\alpha _2$ may affect the sign of the denominator. We first set $\eta _2=0$, so that the denominator is always positive, and thus, we focus only on roots of the numerator.

From inspection, we observe that *B* is a cubic expression in $I^*$ without a constant term. Thus, our cubic expression for the roots of $\frac{\mathrm{{d}}I}{\mathrm{{d}}t}=0$ has the form

10 $$\begin{aligned} 0=I^*[a(I^*)^2+b(I^*)+c], \end{aligned}$$

where

11 $$\begin{aligned} a= & {} -\mu \alpha _2(\beta \eta _1 + \beta \kappa + \beta \mu + \beta \rho + \delta \eta _3), \end{aligned}$$

12 $$\begin{aligned} c= & {} (N^*)^2\mu (\alpha _1\delta \kappa +\alpha _1\delta \mu +\alpha _1\delta \rho + \alpha _1\eta _1\mu +\alpha _1\eta _3\mu \nonumber \\&\quad +\alpha _1\kappa \mu +\alpha _1\mu ^2 +\alpha _1\mu \rho +\delta \kappa \mu + \delta \mu ^2 \nonumber \\&\quad +\delta \mu \rho +\eta _1\mu ^2 +\eta _1\mu \rho +\eta _3\mu ^2 +\eta _3\mu \rho +\kappa \mu ^2+\kappa \mu \omega \nonumber \\&\quad +\mu ^3 +\mu ^2\omega + \mu ^2\rho +\mu \omega \rho )({\mathcal {R}}_0 - 1), \end{aligned}$$

and

13 $$\begin{aligned} b= & {} -\mu N^* (\alpha _1\beta \eta _1 + \alpha _1\beta \kappa + \alpha _1\beta \mu + \alpha _1\beta \rho + \alpha _1\delta \eta _3 - \alpha _2\beta \kappa - \alpha _2\beta \mu - \alpha _2\beta \rho \nonumber \\&+ \alpha _2\delta \kappa \,+ \alpha _2\delta \mu + \alpha _2\delta \rho + \alpha _2\eta _1\mu + \alpha _2\eta _3\mu + \alpha _2\kappa \mu + \alpha _2\mu ^2 + \alpha _2\mu \rho + \beta \eta _1\mu \nonumber \\&+ \beta \eta _1\rho + \beta \kappa \mu \,+ \beta \kappa \omega + \beta \mu ^2 + \beta \mu \omega + \beta \mu \rho + \beta \omega \rho + \delta \eta _3\mu + \delta \eta _3\rho ). \end{aligned}$$

Thus, this *c* term from (10) is positive when ${\mathcal {R}}_0>1$ and it is negative when ${\mathcal {R}}_0<1$. We will use this information to interpret whether or not it is possible to have a backward bifurcation when ${\mathcal {R}}_0<1$ by using Descartes’ Rule of Signs. We know that when ${\mathcal {R}}_0<1$ our *c* term must be negative. We also know that our *a* term in the quadratic in (10) must always be negative. According to Descartes’ Rule of Signs, there can be two or no positive real roots if $b>0$ and no positive real roots if $b<0$. Using our baseline parameters discussed later with the modification that $\delta =.06$ and $\alpha _2=2000$, we observe that $b>0$ and the roots of (10) are positive. This is confirmed in the full system, and thus, we conclude that we can have a backward bifurcation for $\epsilon =\eta _2=0$ for sufficiently large $\alpha _2$ (approximately $>1200$ for the given parameter values). We note before proceeding that the value for $\delta$ is 2 times its current estimated value; in contrast, $\alpha _2=.01$ is the value that fit the data, and thus, the value of nonlinear relapse rate $\alpha _2$ needed for a backward bifurcation is at least 120,000 times greater than this and thus likely unrealistic.

We now keep $\epsilon =0$ and consider $\alpha _2=0$ with $\eta _2>0$. The denominator may become negative for sufficiently large $\eta _2$. Trial and error shows that we can find roots of *B*/*C* that are positive. However, substituting these values into the full system yield negative values for some of the other variables. Following Battista et al. (2019), Castillo-Chavez and Song (2004) as shown in appendix, we show that this case cannot have a backward bifurcation. Thus, without saturation, we can have a backward bifurcation for an unrealistically large $\alpha _2$, the nonlinear relapse from *R* to *I*, but cannot have a backward bifurcation when the nonlinear relapse rate is zero.

Let us now look to analyze the equilibria when $\epsilon > 0$, i.e., we will include the saturation term. We will show that a critical value exists above which a backward bifurcation is permitted. Of particular note is that this critical value for $\epsilon$ is within a reasonable range and the value of $\alpha$ could be 0 or its baseline value.

Proceeding in a more straightforward manner complicates things immediately due to the large algebraic expressions. We tried to simplify the saturation term through a Taylor series expansion for small $\epsilon$ but that approach did not work. Instead, we allow the system, whose total population is governed by $\frac{\mathrm{{d}}N}{\mathrm{{d}}t}=\Lambda -\mu N -\delta I$, to reach its steady-state population level, $N^*$, given by when $N^*=\frac{\Lambda -I\delta }{\mu }$. The resulting limiting system is as follows:

14 $$\begin{aligned} \left\{ \begin{aligned} \frac{\mathrm{{d}}\tilde{S}}{\mathrm{{d}}t}&= \Lambda - \beta S\frac{I\mu }{(\Lambda -I\delta )} -\mu S, \\ \frac{\mathrm{{d}}\tilde{I}}{\mathrm{{d}}t}&= \beta S\frac{I\mu }{(\Lambda -I\delta )}+\alpha _1 R + \alpha _2 R\frac{I\mu }{(\Lambda -I\delta )}+\kappa T\\&~~ - b(T) \left( \eta _1I+\eta _2\frac{IR\mu }{(\Lambda -I\delta )} +\eta _3\frac{IS\mu }{(\Lambda -I\delta )}\right) -(\omega +\mu +\delta )I,\\ \frac{\mathrm{{d}}\tilde{T}}{\mathrm{{d}}t}&= b(T) \left( \eta _1I +\eta _2\frac{R\mu }{(\Lambda -I\delta )}I+\eta _3\frac{S\mu }{(\Lambda -I\delta )} I\right) -(\kappa +\rho +\mu )T,\\ \frac{\mathrm{{d}}\tilde{R}}{\mathrm{{d}}t}&= \omega I +\rho T -(\alpha _1 +\mu )R -\alpha _2R\frac{I\mu }{(\Lambda -I\delta )}, \end{aligned} \right. \nonumber \\ \end{aligned}$$

where $$b(T)=\frac{1}{1+\epsilon T}.$$

We again try to obtain an equation involving only parameters and the variable $I^*$. We proceed as before by solving for $S^*$ by setting $\frac{\mathrm{{d}}\tilde{S}}{\mathrm{{d}}t}=0$ and then plugging that result into $\frac{\mathrm{{d}}\tilde{R}}{\mathrm{{d}}t}=0$ and $\frac{\mathrm{{d}}\tilde{T}}{\mathrm{{d}}t}=0$. Next, we solve for $R^*$ and $T^*$ in terms of $I^*$ simultaneously by setting $\frac{\mathrm{{d}}\tilde{R}}{\mathrm{{d}}t}=0$ and $\frac{\mathrm{{d}}\tilde{T}}{\mathrm{{d}}t}=0$. We plug $S^*,R^*$ and $T^*$ into $\frac{\mathrm{{d}}\tilde{I}}{\mathrm{{d}}t}$. The resulting equation, which we will refer to as ($\star$), is in terms of the variable $I^*$. This is all done using Maple and is not presented here due to its length.

**Fig. 3:**
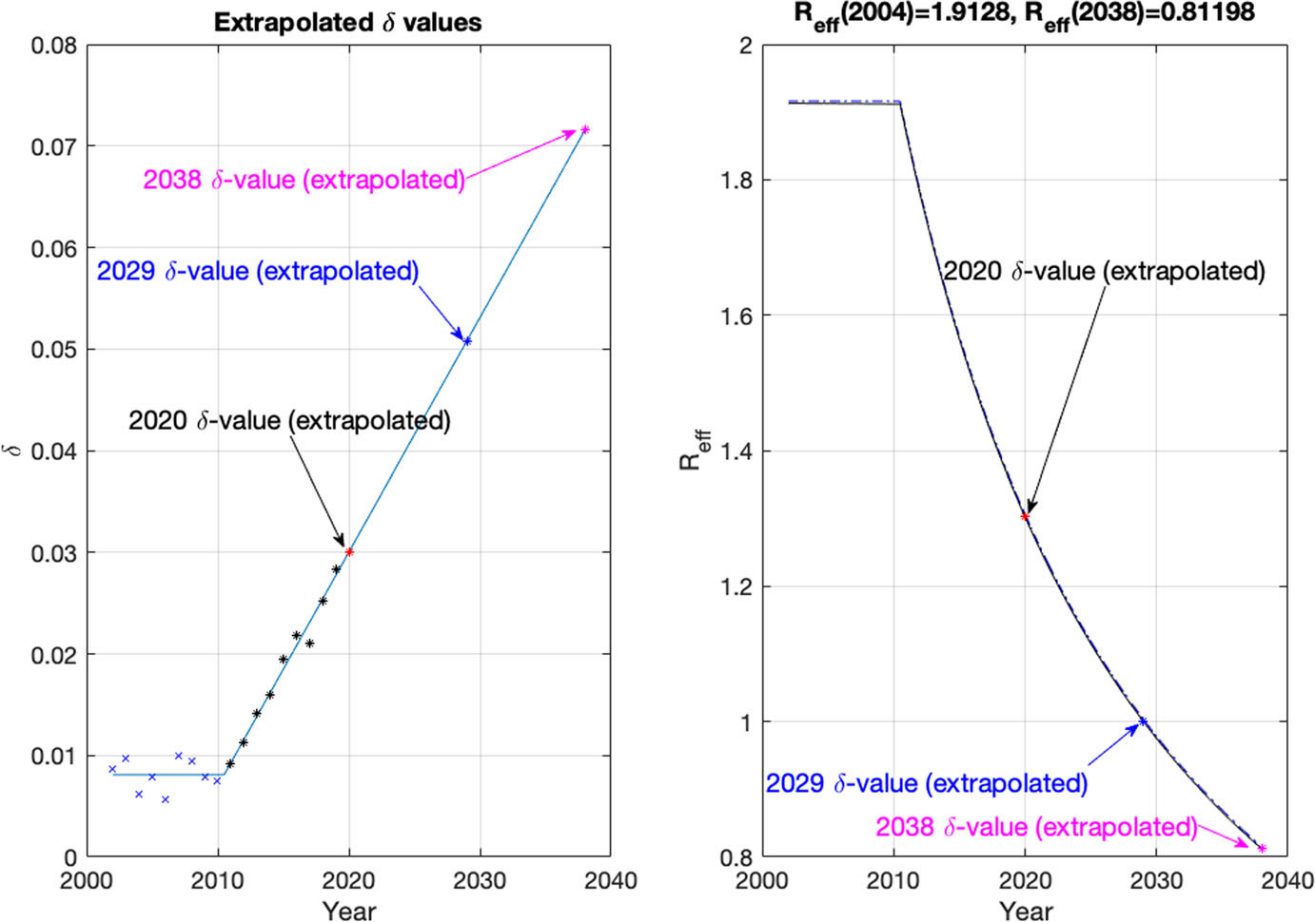
(Left): Extrapolated $\delta$-values. The blue x-marks and black asterisks are from the overdose data and are the same as in the bottom left panel of Fig. 2. The line obtained with a least squares fit of the data from 2011–2019 and given in (4) is extended to 2038. The labeled $\delta$-values in 2020, 2029, and 2038 are from extrapolation (16) using the best fit line. (Right): The effective reproductive number, ${\mathcal {R}}_{{\mathrm{eff}}}(t)=({\mathcal {R}}_0S(t)/N(t))$, is plotted as the solid black curve using the baseline values of the parameters from (5) and the extrapolated $\delta$-values from the best fit line. Just above the ${\mathcal {R}}_{{\mathrm{eff}}}$ curve, ${\mathcal {R}}_0$ is plotted as a dashed blue curve; this close approximation is expected given that $S(0)\approx \Lambda /\mu$ (Color figure online)

Obtaining a general expression for when a backward bifurcation occurred yielded pages of expressions that were too complicated to analyze. We thus chose to focus on three parameters, $\delta$, $\epsilon$, and $\beta$ the parameters addressing overdose death, saturation, and transmission, respectively. We extrapolate the $\delta$-values from Fig. 2 as well as calculate the corresponding effective reproductive number ${\mathcal {R}}_{{\mathrm{eff}}}(t)=({\mathcal {R}}_0S(t)/N_0)$ to determine when the DFE and EE will be stable; see Fig. 3. The results that we now present use realistic parameter values based on data through 2019 and presented in (5) to give stability curves in terms of the overdose death, saturation, and transmission parameters. We can observe regions in the $\delta$–$\epsilon$–$\beta$ parameter space that correspond to the EE stable (only), both DFE and EE stable (bi-stability), and the DFE stable (only). In this latter situation, the EE no longer exists biologically with only the DFE persisting and stable. While this is clearly not a desirable situation, the increase in fentanyl in the heroin supply makes this scenario a potentially realistic one that needs consideration.

**Fig. 4:**
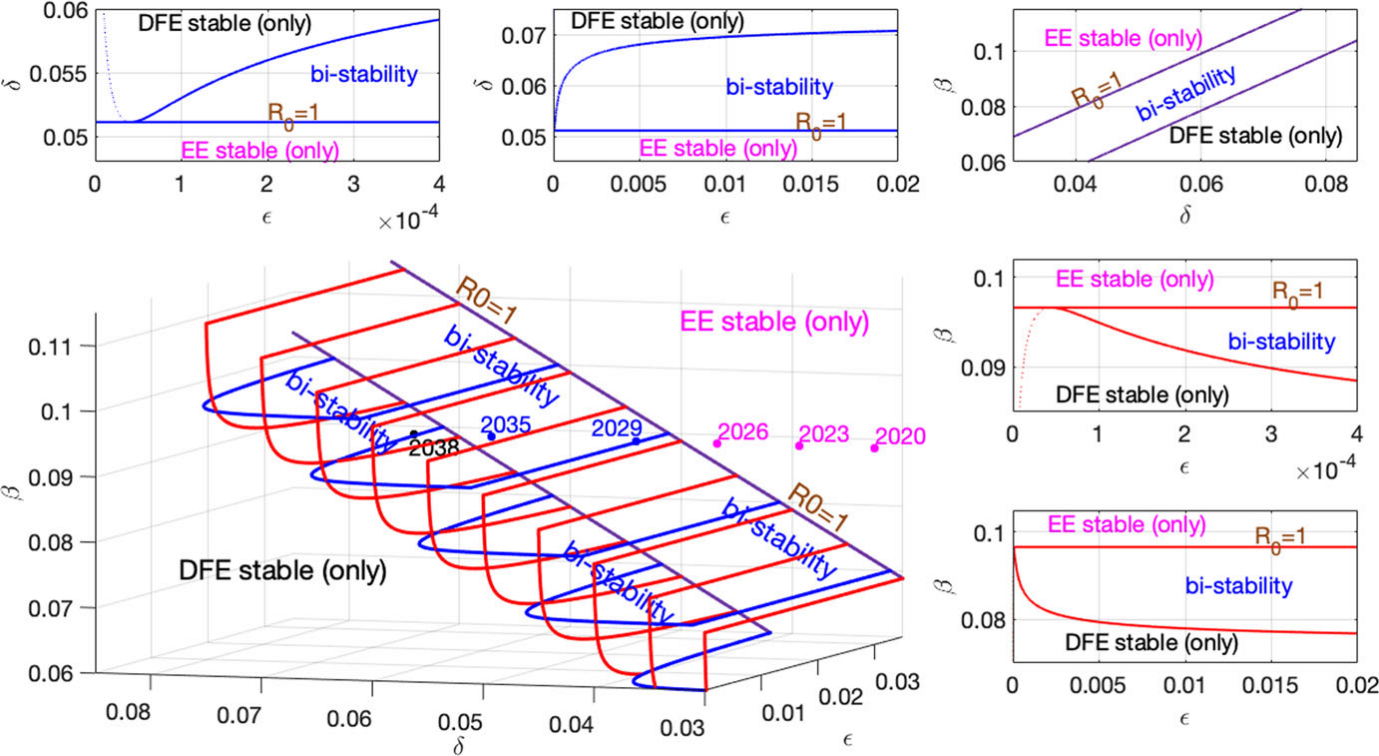
Regions of stability for equilibria. Top panel (left and middle): in the $\epsilon$–$\delta$ plane with $\beta$ fixed at .09, the solid blue horizontal line corresponds to the constant $\delta$ for which ${\mathcal {R}}_0$=1 and below this line only the EE is stable; above this line for large enough $\epsilon$ is the curve that separates the region of bi-stability from where only the DFE is stable. Top panel (right): in the $\delta$–$\beta$ plane with $\epsilon$ fixed at .0313, the two lines separate the regions of (i) EE stable (only), (ii) bi-stability of EE and DFE, and (iii) DFE stable (only); the upper line corresponds to ${\mathcal {R}}_0$=1. Right panel (middle and bottom): In the $\epsilon$–$\beta$ plane with $\delta$ fixed at .0577 (its extrapolated 2032 value), the solid red horizontal line corresponds to the constant $\beta$ for which ${\mathcal {R}}_0$=1 and above this line only the EE is stable; below this line for large enough $\epsilon$ is the curve that separates the region of bi-stability from where only the DFE is stable. Bottom left panel: the previously described curves are put together in the three-dimensional $\delta$–$\epsilon$–$\beta$ space. The dots with years correspond to $\delta$-values from the extrapolated $\delta$-curve with all other parameters fixed at their baseline values from (5) with the color magenta corresponding to EE stable (only), blue corresponding to the region of bi-stability, and black corresponding to DFE stable (only) (Color figure online)

We leave $\delta$, $\epsilon$, and $\beta$ as parameters and substitute the remaining parameter values from (5) into ($\star$) to obtain an equation in the parameters $\delta$, $\epsilon$, and $\beta$ and the variable $I^*$:

15 $$\begin{aligned} 0= & {} I^* [ (I^*)^5\nu _{6}(\delta ,\epsilon ,\beta ) + (I^*)^4 \nu _{5}(\delta ,\epsilon ,\beta ) + (I^*)^3 \nu _{4}(\delta ,\epsilon ,\beta ) + (I^*)^2 \nu _{3}(\delta ,\epsilon ,\beta ) \nonumber \\&\quad + I^* \nu _{2}(\delta ,\epsilon ,\beta ) + \nu _{1}(\delta ,\epsilon ,\beta ) ], \end{aligned}$$

where the coefficients $\nu _{i}(\delta ,\epsilon ,\beta )$ are given in appendix and the subscript refers to the power of $I^*$. We eliminate the variable $I^*$ by simultaneously solving (15) and the derivative of it, thus requiring the condition for a saddlenode bifurcation. This results in a new equation in terms of $\delta$, $\epsilon$, and $\beta$ that is pages of output in Maple. However, we can plot this implicit equation numerically and present this three-dimensional $\delta$–$\epsilon$–$\beta$ surface with five cross-sectional subplots; see Fig. 4. The years given in Fig. 4 correspond with those shown in Fig. 3. For large $\epsilon$, we have the situation where *b*(*T*) is very small, which is not allowing people to go into treatment due to a lack of availability in specialty treatment facilities.

The presence of a backward bifurcation yields a region of bi-stability when ${\mathcal {R}}_{{\mathrm{eff}}}<1$. This means that we will have two asymptotically stable equilibria, the EE and the DFE, and which one a solution approaches simply depends on the initial conditions. Above the plane ${\mathcal {R}}_{{\mathrm{eff}}}=1$ in the 3-d subplot, only the EE is stable. Below this plane, there is a range of parameter values where we may either have bi-stability or have the DFE as the only stable equilibrium.

**Fig. 5:**
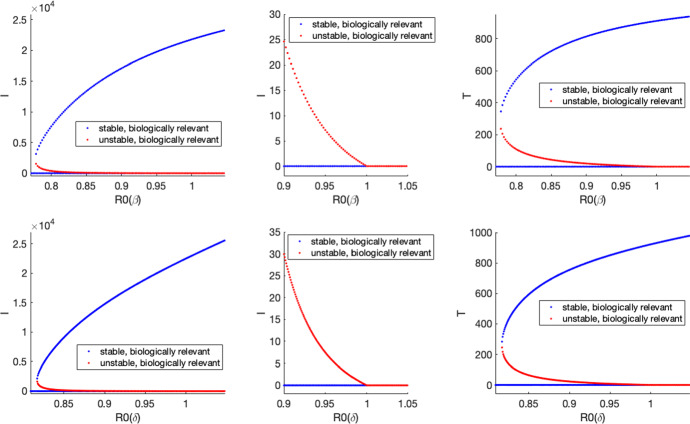
Backward bifurcation plots. The blue curves correspond to stable biologically relevant equilibria and the red curves correspond to unstable biologically relevant equilibria. This demonstrates the difficulty there may be in getting rid of the epidemic once it has taken hold. Top panel: $\delta$ is fixed at .0531, its extrapolated 2030 value; $\beta$ is varied to change ${\mathcal {R}}_0$. Bottom panel $\beta$ is fixed at .09; $\delta$ is varied to change ${\mathcal {R}}_0$ with $\delta \approx .051$ (2029 on its extrapolated curve) corresponding to ${\mathcal {R}}_0=1$. All other parameter values are from (5). The middle column differs from the first column in scale only (Color figure online)

For a given set of parameters, there is a critical $\epsilon _c>0$ that is required for bi-stability and a backward bifurcation. There is an inverse relationship between the saturation term, $\epsilon$, and an availability of specialty treatment facilities. Thus, a lack of availability of specialty treatment facilities that occurs when $\epsilon _c>0$ can give rise to a situation in which the epidemic persists even though the conditions are such that ${\mathcal {R}}_0<1$. See Fig. 5.

###  Sensitivity Analysis

For our sensitivity analysis, we run the model from 2002 to 2020 using the parameters in (4)–(5) and then use the resulting 2020 model output values as our initial conditions. We use the baseline parameters given in (5) that generated this data match and consider two scenarios for $\delta$: (i) assume that $\delta (t)=\delta (2020)=.03002$ for $t\ge 2020$, which we interpret as the fentanyl levels being kept at their 2020 levels, and (ii) assume that $\delta$ is defined by extrapolation based on its least squares fit line given in (16), which we interpret as the fentanyl levels in the heroin supply increasing. In both cases, we consider what happens in 2030 for the sensitivity. In the first scenario, $\delta$ is a constant and will be a parameter in our sensitivity analysis. For the second scenario, we explicitly rewrite $\delta (t)$ in (4) in a form that allows for a $\pm 10\%$ vertical shift at 2020 as well as a potential shift in the slope of the line by $\pm 10\%$ at 2020. This is accomplished via the extension of the least squares fit line in (4), shown in the left panel of Fig. 3, with $m,b>0$ and written as

16 $$\begin{aligned} \delta (t)= & {} m \cdot (t-2020) - b\cdot \left( 1-\frac{0.002307120199666}{4.630363924326326} \cdot 2020\right) , \qquad t \ge 2020, \nonumber \\= & {} m \cdot (t-2020) + b\cdot (0.006483049602509) , \qquad t \ge 2020, \end{aligned}$$

where a percent change in *b* changes $\delta$ through a vertical shift by the same percent change of its 2020 value and a percent change in *m* changes the slope of $\delta$ by the same percent change. With baseline values of $m=0.002307120199666$ and $b=4.630363924326326$ from (4), we will examine these 2 additional parameters in our sensitivity analysis for the second scenario (McLeod et al. 2006).

In order to determine the sensitivity of the system to the input parameters, we perform a sensitivity analysis using partial rank correlation coefficient (PRCC) method (Marino et al. 2008). The PRCC method only applies when there is a monotonic relationship between the model parameters and the output values against which sensitivity is measured. We performed monotonicity checks for all our parameter and initial values and concluded that there is a monotonic relationship.

For our system, we consider the parameter values obtained through parameter estimation and given in (5) as the baseline parameter values. When we consider the extrapolated function for $\delta (t)$, we observe from Fig. 4 that the value $\delta (2030)$ puts the system in the region of bi-stability.

We let the parameters and initial conditions vary $\pm 10\%$ from their baseline values in 2020.

### Discussion of the PRCC Values

We present the sensitivity of our variables *S*, *I*, *T*, and *R* to the parameters of the system in plots and tables in appendix and focus here on variables that may be of more interest to healthcare professionals and policy makers: number of those entering *I* (HUD) for the first time (yearly new HUD), the yearly number of relapses from *T*, the yearly number of relapses from *R* and heroin-related deaths.

While none of these are the variables in our original system, all can be calculated by keeping track of components that contribute to changes in our model variables.

We consider two graphs for each case corresponding to the sensitivities in 2030 for the constant death rate ($\delta = 0.03002$) vs. the variable death rate (16).

In describing the sensitivity results we will refer to a PRCC value of 0.85 or higher as “highly significant,” a PRCC value of 0.70–0.84 as “significant,” values of 0.55–0.69 “somewhat significant,” values of 0.45–0.54 as “slightly significant,” values of .40–0.44 as “borderline significant,” and under .40 as “not significant.”

As can be seen in the tables, some of the initial conditions may show up as significant or highly significant. We fit the 2002–2019 data to baseline parameters with the model output final (year 2020) values forming the initial conditions for our PRCC analysis. While *S*(0), *I*(0), *T*(0), and *R*(0) cannot really be changed, having somewhat different data (e.g., more accurate data) could represent the importance of their significance. Additionally, for the parameter $\mu$ (the natural death rate of the general population), regardless of its significance, it is not a parameter that can be altered since it is the natural death rate. Therefore, we would not focus on it either because it is something we do not have control over. For the following, only the parameters that we have control over will be discussed.

For the following variables’ PRCC results that will be discussed, it can be seen that the graphs at the end time of 2030 are similar for the constant death rate ($\delta = 0.03002$) vs. the variable death rate (16). However, the PRCC values of the parameters are equal to or lower in magnitude at the end time of 2030 for the constant death rate than for the variable death rate. This could be due to the fact that the variable death rate results in higher number of deaths, which has the effect of lowering the HUD class. We always want to lower the number of individuals in the HUD class in beneficial ways. However, with the higher death rate it becomes more crucial for individuals to exit out of the HUD class quicker due to the increased risk of heroin-related overdose. If the treatment rates and/or recovery rates could be increased and more users leave the HUD class and enter treatment, they would be protected from those resulting dangers that could lead to a heroin-related overdose death. It is vital at the higher death rates to get individuals out of the HUD class quicker than for the lower death rate.

**Fig. 6:**
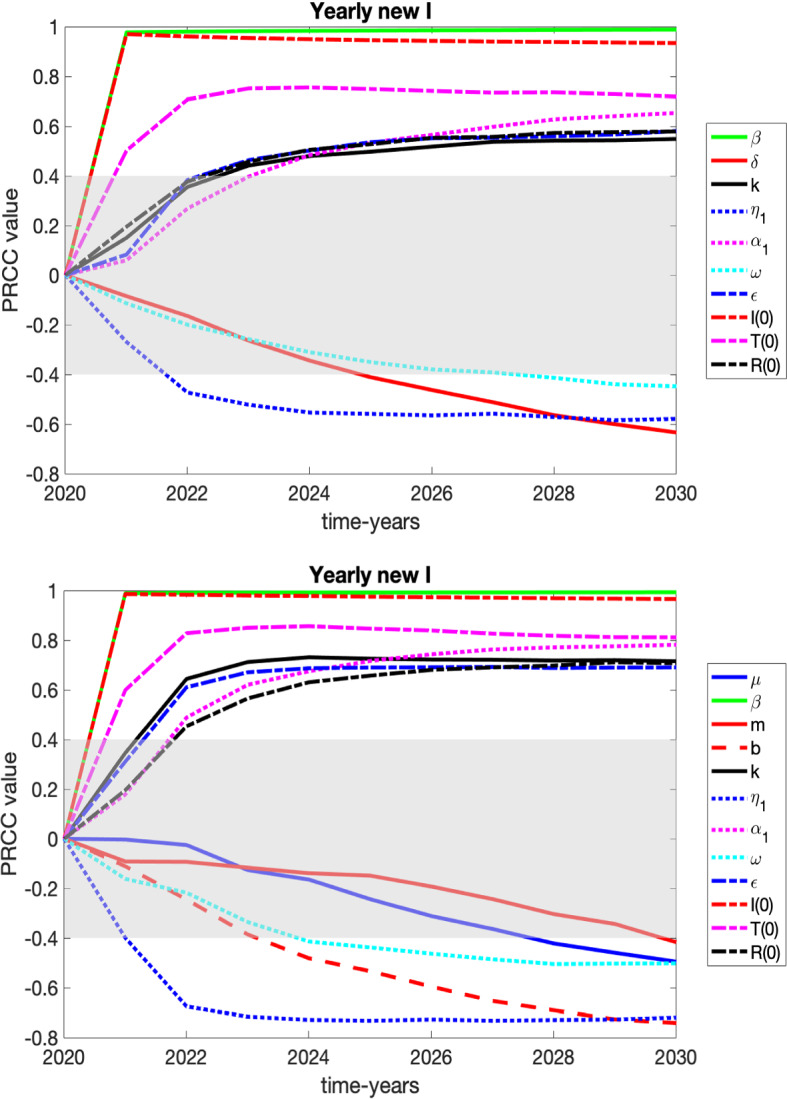
PRCC results over time for those who are entering *I* (HUD) for the first time, with grayed region denoting a lack of significance. These results are summarized in the text and in Table 3. Top: constant death rate of $\delta =.03002$, its extrapolated 2020 value. Bottom: variable death rate defined in (16) (Color figure online)

**Table 3:** PRCC results for movement into *I* (HUD) using baseline parameters (5) and either constant $$\delta =0.03002$$ or the variable $$\delta $$ in (16). The initial conditions for $$t=0$$ in 2020 were generated using (4), (5), 2002 values of $$S= 199{,}500$$, $$I=102$$, $$T= 95 $$, $$R= 100$$, and running the system until 2020 (as previously described to obtain Fig. 2). The PRCC values at 2030 are given here with the columns labeled “constant” corresponding to the constant death rate of $$\delta = 0.03002$$ (its extrapolated 2020 value) and the columns “variable” corresponding to the variable death rate defined in (16)

IC/ param	Yearly new *I*		Yearly relapse from *T*		Yearly relapse from *R*		Yearly deaths	
	Constant	Variable	Constant	Variable	Constant	Variable	Constant	Variable
*S*(0)	–	–	–	–	–	–	–	–
*I*(0)	0.93	0.97	0.85	0.89	0.94	0.95	0.95	0.96
*T*(0)	0.72	0.81	0.50	0.62	0.76	0.78	0.69	0.79
*R*(0)	0.58	0.71	–	0.50	0.78	0.79	0.62	0.68
$\Lambda \ *$	–	–	–	–	–	–	–	–
$\mu$	–	$-$ 0.49	–	–	$-$ 0.45	$-$ 0.54	$-$ 0.40	$-$ 0.52
$\beta$	0.99	0.99	0.79	0.85	0.85	0.88	0.93	0.95
$\eta _1$	$-$ 0.58	$-$ 0.72	0.78	0.81	–	–	$-$ 0.65	$-$ 0.75
$\eta _2 \ *$	–	–	–	–	–	–	–	–
$\eta _3$	–	–	0.39	0.40	–	–	$-$ 0.29	–
$\rho$	–	–	$-$ 0.47	$-$ 0.56	0.89	0.90	–	–
$\kappa$	0.55	0.71	0.94	0.96	–	$-$ 0.41	0.60	0.72
$\alpha _1$	0.65	0.78	–	0.54	0.89	0.90	0.67	0.76
$\alpha _2 \ *$	–	–	–	–	–	–	–	–
$\delta$	$-$ 0.63	–	$-$ 0.45	–	$-$ 0.53	–	0.96	–
*m*	–	$-$ 0.42	–	–	–	–	–	0.85
*b*	–	$-$ 0.74	–	$-$ 0.52	–	$-$ 0.49	–	0.87
$\omega$	$-$ 0.45	$-$ 0.50	–	–	0.90	0.92	$-$ 0.51	$-$ 0.58
$\epsilon \ *$	0.58	0.69	$-$ 0.75	$-$ 0.82	–	–	0.65	0.64

**Fig. 7:**
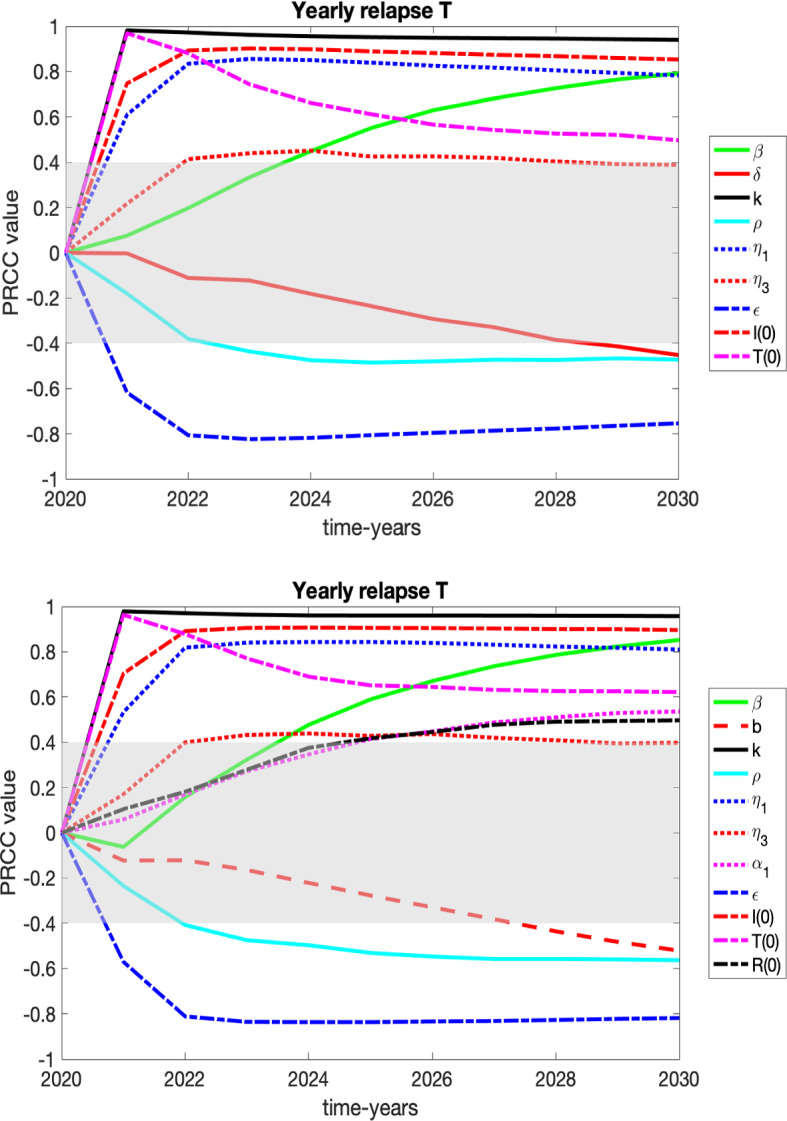
PRCC results over time for those who are entering *I* (HUD) by relapsing from *T*, with grayed region denoting a lack of significance. These results are summarized in the text and in Table 3. Top: constant death rate of $\delta = 0.03002$, its extrapolated 2020 value. Bottom: variable death rate defined in (16) (Color figure online)

**Fig. 8:**
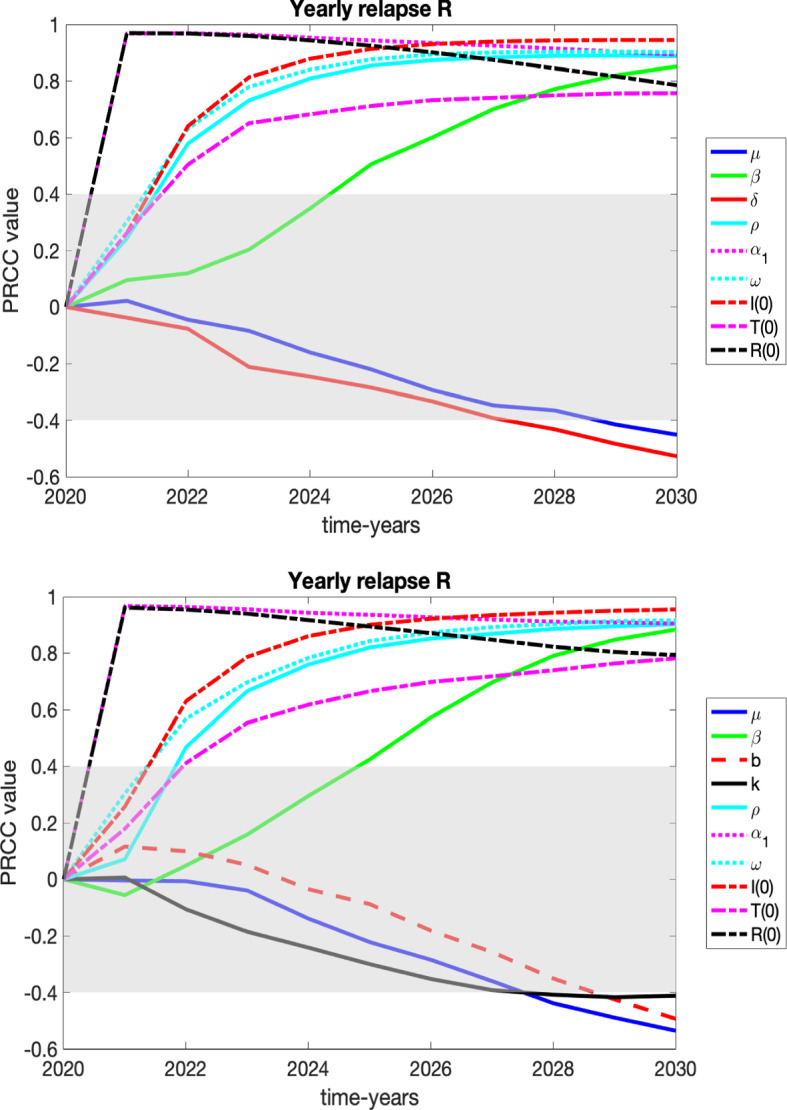
PRCC results over time for those who are entering HUD by relapsing from *R*, with grayed region denoting a lack of significance. These results are summarized in the text and in Table 3. Top: constant death rate of $\delta = 0.03002$, its extrapolated 2020 value. Bottom: variable death rate defined in (16) (Color figure online)

**Fig. 9:**
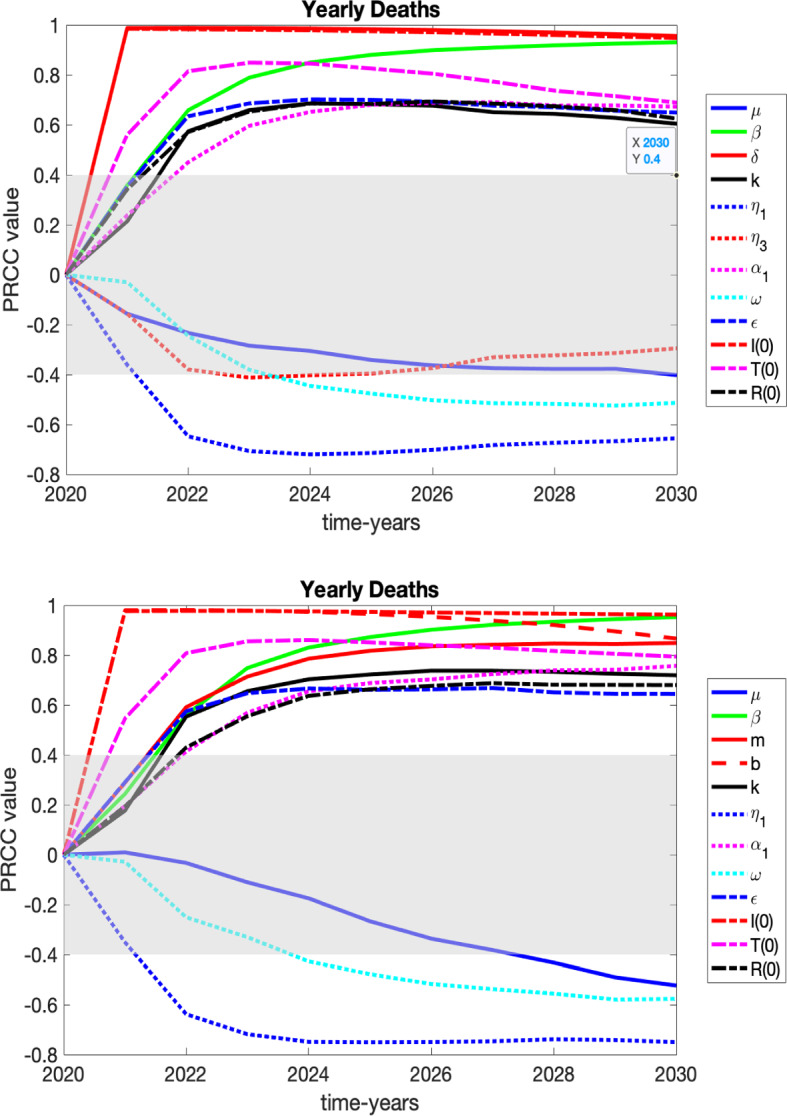
PRCC results over time for the yearly HUD overdose deaths, with grayed region denoting a lack of significance. These results are summarized in the text and in Table 3. Top: constant death rate of $\delta = 0.03002$, its extrapolated 2020 value. Bottom: variable death rate defined in (16) (Color figure online)

All table entries without a value are not significant. The notation of * denotes that the parameter does not appear in the formula for ${\mathcal {R}}_0$. The corresponding graphs for this table are given in Figs. 6, 7, 8, 9

$${\text {Yearly new}} \,I:$$

The yearly new *I* variable keeps track of the number of individuals from the *S* class who are entering the *I* (HUD) class; see Fig. 6 and Table 3. The comparisons of the PRCC values for the yearly deaths due to overdose at the year-end time of 2030 were very similar for both death rates. What follows discusses both death rates unless otherwise noted. The parameter with the highest significance (ranked highly significant for both death rates) to focus on would be $\beta$ (the transmission rate of becoming an individual with HUD through interaction with others in the HUD class). Since this parameter is positively correlated, a decrease of the transmission rate would result in a decrease of the yearly new to HUD counts, as expected. Although not as significant as the transmission rate, but ranked somewhat significant to significant, other parameters to consider for focus would be $\alpha _1$ (the rate of individuals in the recovered state relapsing back to the HUD class on their own), $\epsilon$ (saturation term for entering treatment), $\kappa$ (the rate of individuals leaving treatment and returning to the HUD class), $\eta _1$ (the rate of individuals in HUD who enter a specialty treatment facility on their own), and $\delta$ (death rate of individuals in the HUD class due to overdose). Thus, decreasing the relapse rate from treatment and the recovered class, increasing availability of specialty treatment, or increasing the rate of access for someone to enter treatment on their own would all decrease the yearly new to HUD counts. Although an increase in the HUD death rate would decrease the counts, as expected since less individuals remaining in HUD would result in less of the *S* class moving to HUD, ethically, we do not want the counts to decrease because of less interactions due to the high death rate. Therefore, the previously mentioned parameters, other than the HUD death rate, are best to focus on.

$${{\textit{Yearly relapse }}T:}$$

The Yearly relapse *T* variable keeps track of the number of individuals who were in treatment and have relapsed back into the HUD class; see Fig. 7 and Table 3. The comparisons of the PRCC values for the yearly deaths due to overdose at the year-end time of 2030 were very similar for both death rates. What follows discusses both death rates unless otherwise noted. The parameter with the highest significance (ranked highly significant) to focus on would be $\kappa$ (the rate of individuals leaving treatment and returning to the HUD class). Since $\kappa$ is positively correlated, a decrease in the relapse rate of treatment decreases the yearly relapse *T* counts, as expected. Other parameters that followed in significance (ranked somewhat significant to significant) are $\beta$ (the transmission rate of becoming an individual with HUD through interaction with others in the HUD class), $\eta _1$ (the rate of individuals in HUD who enter a specialty treatment facility on their own), and $\epsilon$ (saturation term for entering treatment). Thus, decreasing the transmission rate, increasing the rate of individuals entering treatment by one’s own accord, and increasing availability of treatment would all decrease the yearly relapse *T* counts.

$${{\textit{Yearly relapse }}R:}$$

The yearly relapse *R* variable keeps track of the individuals who were in the *R* class and have relapsed back into the *I* (HUD) class whether on their own or by being in contact with someone from the HUD class; see Fig. 8 and Table 3. The comparisons of the PRCC values for the yearly deaths due to overdose at the year-end time of 2030 were very similar for both death rates. What follows discusses both death rates unless otherwise noted. The parameter with the highest significance (ranked highly significant) is $\omega$ (the rate of individuals in HUD who enter the recovered class by either treatment in non-specialty facilities and/or “quitting cold turkey”). Since $\omega$ is positively correlated, a decrease in the number of people entering the recovered class decreases the number of yearly relapse *R* counts; however, we do not want the count to decrease by lowering the rate individuals go into recovery. Hence, we look at the next most significant parameters (ranked highly significant) which are $\rho$ (the rate of individuals leaving treatment and entering a “recovered” state), $\alpha _1$ (the rate of individuals in the recovered state relapsing back to the *A* class on their own), and $\beta$ (the transmission rate of becoming an individual with HUD through interaction with others in the HUD class). Similar to the analysis for $\omega$, we ignore decreasing $\rho$ to decrease the counts, and thus, the most significant parameters to focus on would be $\beta$ and $\alpha _1$. Hence, decreasing the transmission rate and/or decreasing the relapse rate of individuals from *R* to HUD would decrease the yearly relapse *R* counts.

*Yearly deaths:*

The yearly deaths variable accounts for the number of yearly deaths due to overdose by HUD individuals; see Fig. 9 and Table 3. The comparisons of the PRCC values for the yearly deaths due to overdose at the year-end time of 2030 were very similar for both death rates. What follows discusses both death rates unless otherwise noted. The two most significant parameters (ranked highly significant) are the death rates, $\delta$ (death rate of individuals in the HUD class due to overdose)(scenario (i)), *b* and *m* (scenario (ii)), and $\beta$ (the transmission rate of becoming an individual with HUD through interaction with others in the HUD class). Hence, lowering the HUD death rate and transmission rate would decrease the yearly death counts, as expected. Other parameters (ranked somewhat significant to significant) are $\alpha _1$ (the rate of individuals in the recovered state relapsing back to the HUD class on their own), $\epsilon$ (saturation term for entering treatment), $\kappa$ (the rate of individuals leaving treatment and returning to the HUD class), $\eta _1$ (the rate of individuals in HUD who enter a specialty treatment facility on their own), and $\omega$ (the rate of individuals in HUD who enter the recovered class by either treatment in non-specialty facilities and/or “quitting cold turkey”). Therefore, lowering the relapse rates from treatment and the recovered class, increasing availability for treatment, increasing the rate of number of individuals entering treatment on their own, and/or increasing the rate of individuals entering the recovered class would all result in decreasing the yearly deaths.

## Conclusion

Our paper presents a deterministic model for the dynamics of an illicit opioid use disorder (IOUD) model. Besides a traditional susceptible class and a class of individuals with illicit OUD, our model includes a treatment class for individuals in specialty treatment facilities. It further includes a recovered population class that holds individuals who have either completed treatment (specialty or non-specialty) or “quit cold turkey.” Here, they may remain or relapse back to the IOUD class. Our model also includes a saturation treatment function, which slows down the rate of entry into treatment due to the lack of availability of specialty treatment. Realistic parameter estimates are obtained from the literature and via parameter estimation to match the available SAMHSA data from 2002–2019. The overdose death rate for those in the IOUD class is seen to have been increasing at a linear rate since around 2011. In addition, since our model approaches a constant population $N^*=(\Lambda -I^*\delta )/\mu$, scaling the SAMHSA data to a population of 200,000 allows us to better see the dynamics of this heroin epidemic.

For the parameter estimates we found, the $\delta$-value extrapolated to 2030 results in a situation where the effective reproductive number, ${\mathcal {R}}_{{\mathrm{eff}}}$, is less than one, yet a region of bi-stability exists in the $\delta$–$\epsilon$–$\beta$ space in which both EE and DFE are stable. There is a backward bifurcation that occurs just below ${\mathcal {R}}_{{\mathrm{eff}}}=.82$ as $\delta$ is varied (for fixed $\beta =.09$) and just below ${\mathcal {R}}_{{\mathrm{eff}}}=.78$ as $\beta$ is varied (for fixed $\delta =.0531$) illustrating an additional difficulty of eradicating HUD. This region of bi-stability predicts a minimum $\epsilon$-value below which we will not have bi-stability. Thus, ensuring adequate access to specialty treatment facilities is important. In addition, while our model has a backward bifurcation for no saturation, it requires an unrealistically large nonlinear relapse rate $\alpha _2$; in contrast, with saturation, a backward bifurcation exists above the minimum $\epsilon$-value for a realistic nonlinear relapse rate (including a value of $\alpha _2 = 0$).

A surprising discovery in our analysis was that if the growth of the illicit OUD overdose death rate continues on its path of the last 10 years, by 2038 the DFE will be the only stable biologically relevant equilibrium. While we do want this epidemic to end, we do not want it to end because of overdose deaths from illicit opioid use. Law enforcement intervention, policies, and/or strategies can be taken to either slow the increase of $\delta$, keep the rate constant, or possibly reduce it.

While many of the results of our sensitivity analysis were expected, one result stood out—the consistent importance of $\eta _1$, which is the parameter quantifying the rate at which someone in HUD enters *T* on their own accord. Out of the three variables to move into treatment, $\eta _1$ was more important than $\eta _2$, entering treatment because of interaction with a susceptible, or $\eta _3$, entering treatment because of an interaction with a recovered person. It would seem beneficial in the short term to increase efforts for ways that make it easier for an individual to enter treatment if needed. This could be through things such as financial support for treatment or perhaps lowering the stigma to increase willingness to seek out help on their own as well.

Future work could include extensions to the model such as incorporating a prescription class, a “casual user” class, or a second treatment class for non-specialty. Finally, parameter estimation revealed the necessity of additional statistics that could be calculated and questions that could be asked by SAMHSA in their NSDUH that would allow for a better comparison of model output to data, including calculating heroin use disorder (HUD) in the last month and determining synthetic opioids use with all the time frames given including “in the last month.” Keeping track of whether those individuals in treatment came from the *I* class or from a “casual user” class would also help in estimating parameters.

Final notes: During the revisions of this paper, the SAMHSA data for 2020 were released. We observe that there was a change in the definition of individuals with substance use disorder (SUD), including HUD, due to the switch in criteria for classifying these individuals. “Beginning with the 2020 NSDUH, SUD estimates for alcohol and illicit drugs were based on criteria in the *Diagnostic and Statistical Manual of Mental Disorders*, 5th edition,” where previously the 4th edition was used (Center for Behavioral Health Statistics and Quality 2021). Due to the different definition for classifying HUD, we cannot directly incorporate the new data into our model and leave it to future work to determine how to incorporate it.

## Figures and Tables

**Fig. 10:**
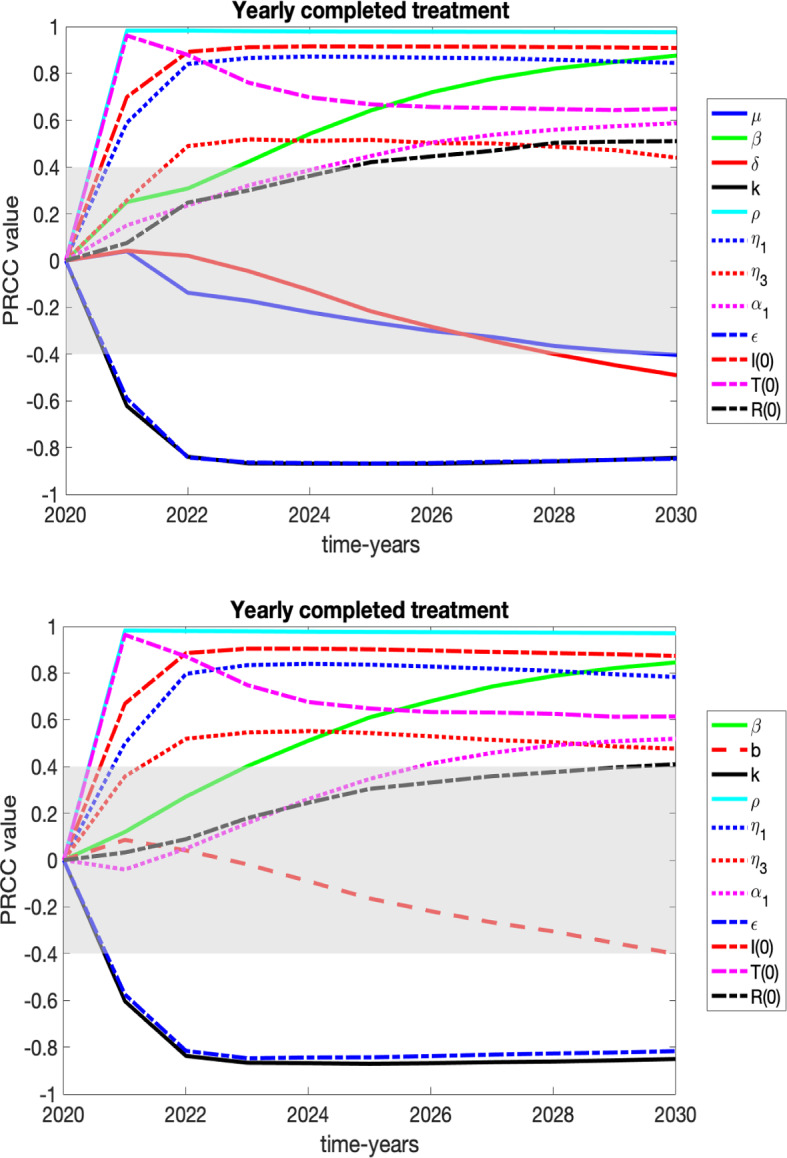
PRCC results over time for the model variable yearly completed treatment variable (those individuals who were in treatment and have moved into the recovered class), with grayed region denoting a lack of significance. These results are summarized in Table 4. Top: constant death rate of $\delta = 0.03002$, its extrapolated 2020 value. Bottom: variable death rate defined in (16) (Color figure online)

**Fig. 11:**
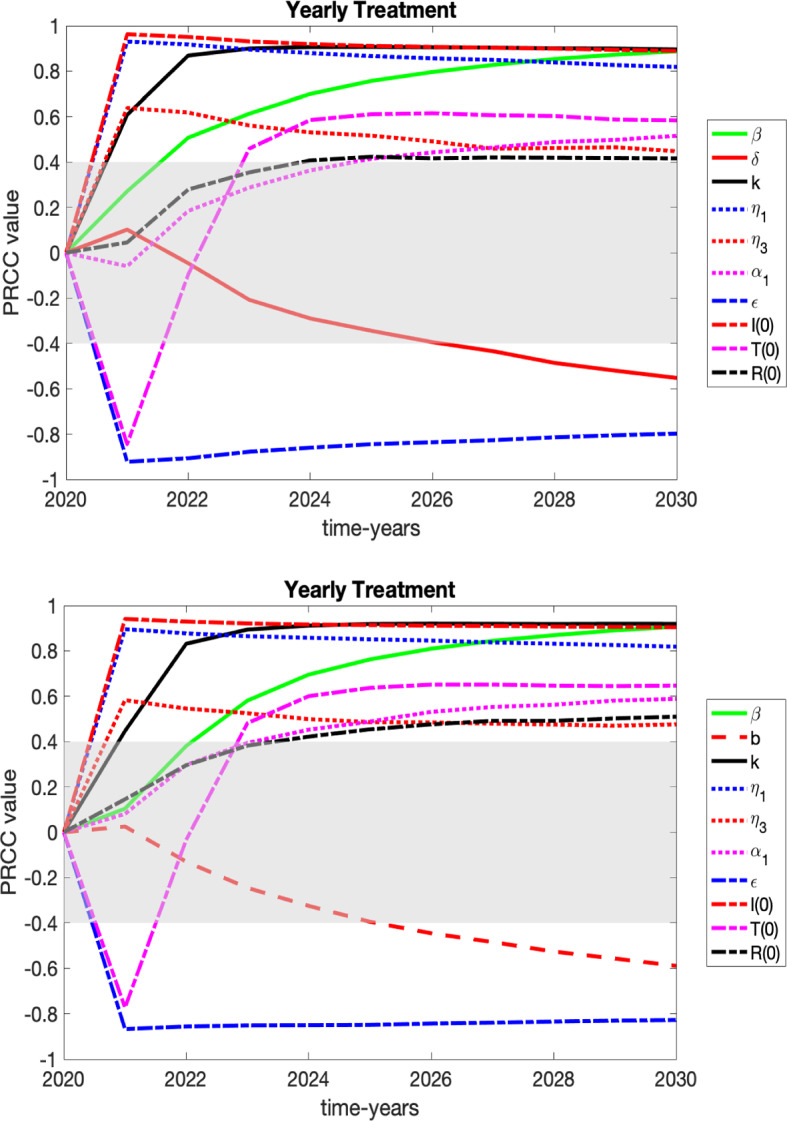
PRCC results over time for the Yearly treatment variable (those individuals who went to treatment), with grayed region denoting a lack of significance. These results are summarized in Table 4. Top: constant death rate of $\delta = 0.03002$, its extrapolated 2020 value. Bottom: variable death rate defined in (16) (Color figure online)

**Fig. 12:**
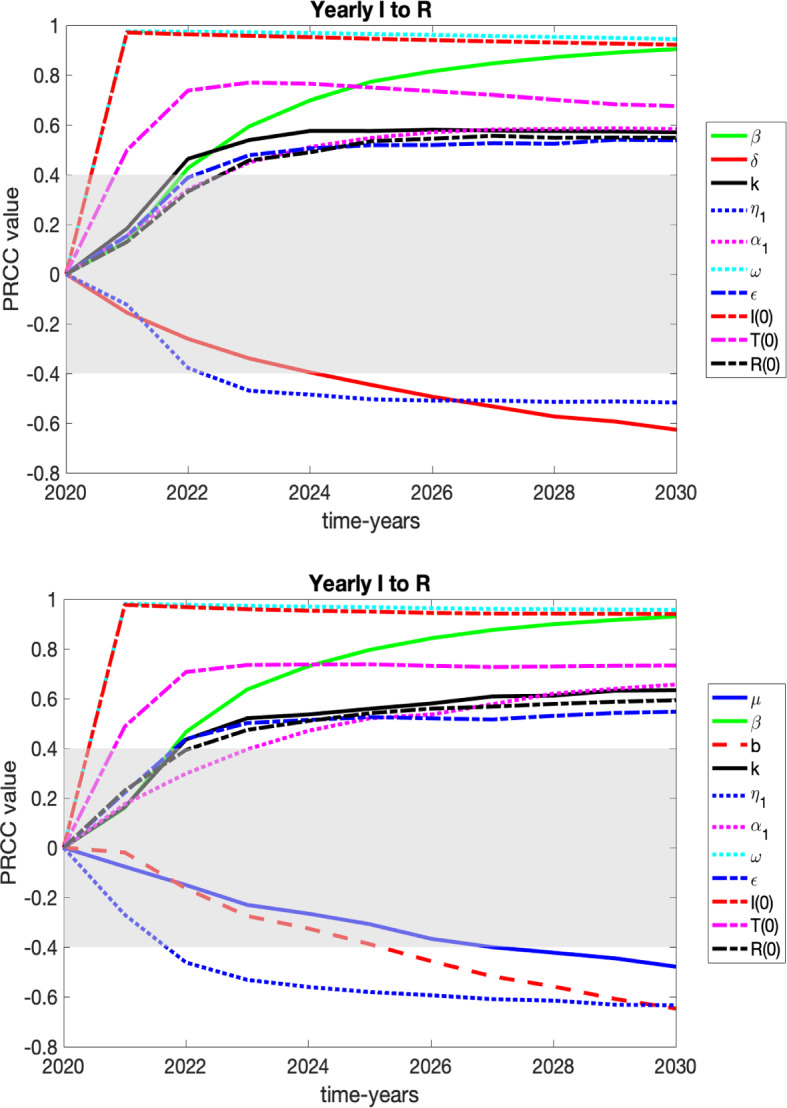
PRCC results over time for Yearly *I* to *R* (those who left the IOUD class either quitting on their own or with the help of a non-specialty treatment facility), with grayed region denoting a lack of significance. These results are summarized in Table 4. Top: constant death rate of $\delta = 0.03002$, its extrapolated 2020 value. Bottom: variable death rate defined in (16) (Color figure online)

**Fig. 13:**
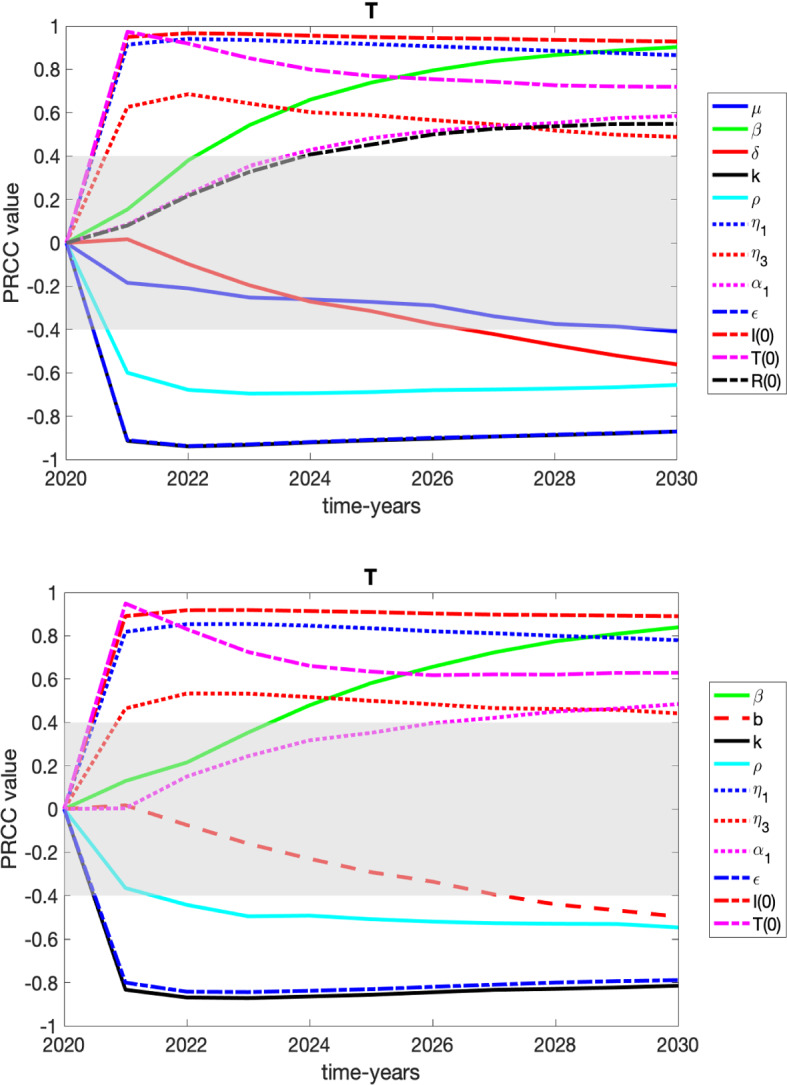
PRCC results over time for the model variable *T*, with grayed region denoting a lack of significance. These results are summarized in Table 5. Top: constant death rate of $\delta = 0.03002$, its extrapolated 2020 value. Bottom: variable death rate defined in (16) (Color figure online)

**Fig. 14:**
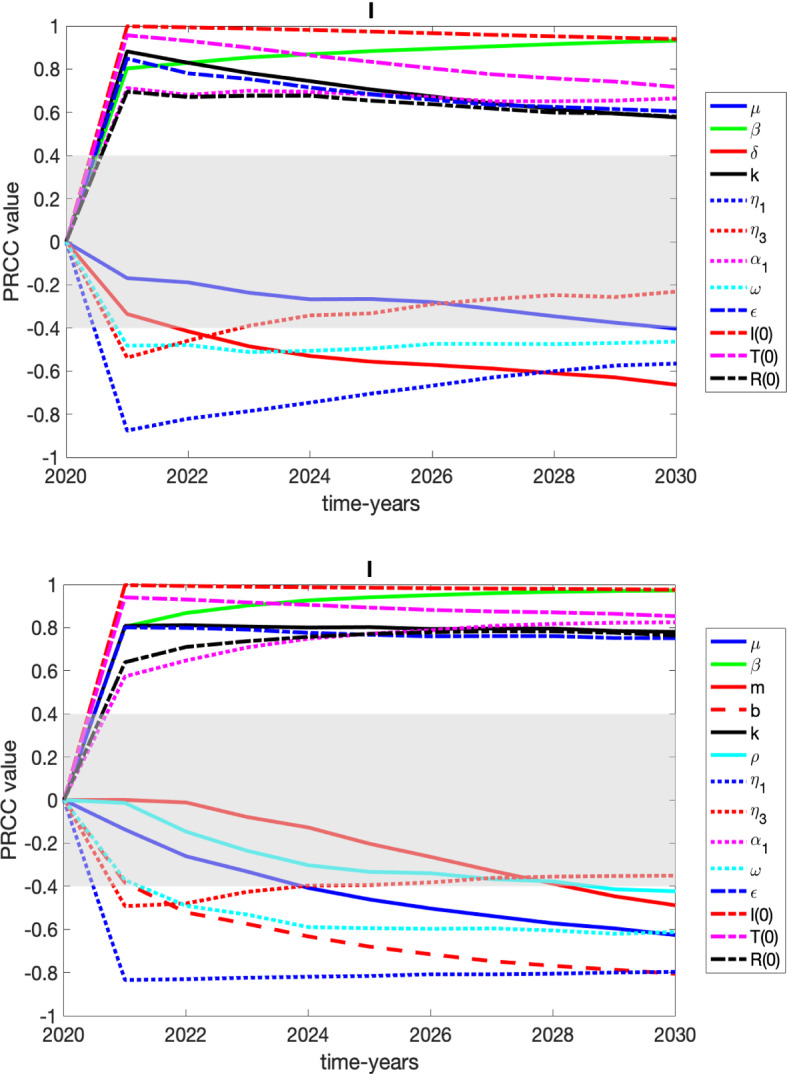
PRCC results over time for the model variable *I*, (HUD), with grayed region denoting a lack of significance. These results are summarized in Table 5. Top: constant death rate of $\delta = 0.03002$, its extrapolated 2020 value. Bottom: variable death rate defined in (16) (Color figure online)

**Fig. 15:**
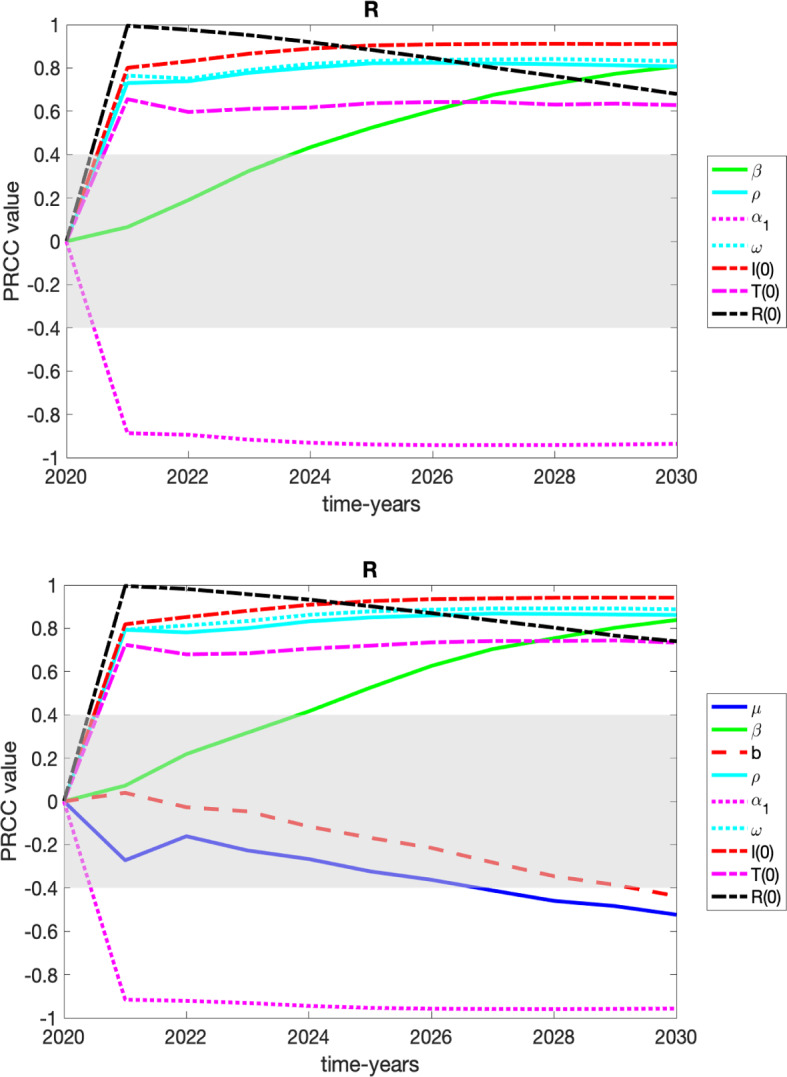
PRCC results over time for the model variable *R*, with grayed region denoting a lack of significance. These results are summarized in Table 5. Top: constant death rate of $\delta = 0.03002$, its extrapolated 2020 value. Bottom: variable death rate defined in (16) (Color figure online)

## Appendix

### Basic Properties

In this section, the basic dynamical features of the illicit opioid use disorder (IOUD) model will be explored.

Since $N(t)=S(t)+I(t)+T(t)+R(t)$, we have $\frac{\mathrm{{d}}N(t)}{\mathrm{{d}}t}=\frac{\mathrm{{d}}S(t)}{\mathrm{{d}}t}+\frac{\mathrm{{d}}I(t)}{\mathrm{{d}}t}+\frac{\mathrm{{d}}T(t)}{\mathrm{{d}}t}+\frac{\mathrm{{d}}R(t)}{\mathrm{{d}}t}$.

Adding the four equations of (1), the total population dynamics are driven by the following differential equation:

17 $$\begin{aligned} \frac{\mathrm{{d}}N}{\mathrm{{d}}t}= & {} \frac{\mathrm{{d}}S}{\mathrm{{d}}t}+\frac{\mathrm{{d}}I}{\mathrm{{d}}t}+\frac{\mathrm{{d}}T}{\mathrm{{d}}t}+\frac{\mathrm{{d}}R}{\mathrm{{d}}t}\nonumber \\= & {} \Lambda -\mu S -(\mu +\delta ) I -\mu T -\mu R\nonumber \\= & {} \Lambda -\mu N -\delta I \end{aligned}$$

Since the IOUD model tracks human populations, all of the associated parameters are nonnegative.

#### Theorem 1

Local solutions to the IOUD model with initial data in the region

$$\begin{aligned} \Omega= & {} \{(S,I,T,R)\in {\mathbb {R}}_+^4:0< S,0<I,0< T,0<R\}, \\ S(0)= & {} S_0> 0, I(0)=I_0> 0, T(0)=T_0> 0, R(0)=R_0>0, N(0)=N_0>0. \end{aligned}$$

exist and are unique.

#### Proof

Let us consider the set $\Omega$ and initial conditions for the system (1):

$$\begin{aligned} \Omega= & {} \{(S,I,T,R)\in {\mathbb {R}}_+^4:0< S,0<I,0< T,0<R\},\\ S(0)= & {} S_0> 0, I(0)=I_0> 0, T(0)=T_0> 0, R(0)=R_0>0, N(0)=N_0>0. \end{aligned}$$

It is easy to see that the functions contained in (1) are differentiable, which ensures its solutions with positive initial values exist and are unique by a direct application of standard differential equation theory (Perko 2013). $\square$

#### Theorem 2

Given nonnegative initial conditions and parameter values, solutions to the IOUD model are nonnegative on the interval of existence.

#### Proof

(i) To verify the $\frac{\mathrm{{d}}S}{\mathrm{{d}}t}$ equation satisfies the conditions of Proposition A.1 in *Mathematics in Population Biology* by Horst Thieme, Thieme (2018), let $S=0$. Then
  18 $$\begin{aligned} \frac{\mathrm{{d}}S}{\mathrm{{d}}t}= & {} \Lambda - \beta \underbrace{S}_{=0}\frac{I}{N} -\mu \underbrace{S}_{=0}\nonumber \\= & {} \Lambda \end{aligned}$$
(ii) To verify the $$\frac{\mathrm{{d}}I}{\mathrm{{d}}t}$$ equation satisfies the conditions of Proposition A.1 (Thieme 2018), let $$ I=0$$, and assume $$ T, R\ge 0$$. Then
  19 $$\begin{aligned} \frac{\mathrm{{d}}I(t)}{\mathrm{{d}}t}= & {} \beta S\frac{\overbrace{I}^{=0}}{N}+\alpha _1 \underbrace{R}_{\ge 0}+ \alpha _2 R\frac{\overbrace{I}^{=0}}{N} +\kappa \underbrace{T}_{\ge 0} \nonumber \\&-b(T)(\eta _1 \underbrace{I}_{= 0}+\eta _2\frac{R}{N} \underbrace{I}_{= 0}+\eta _3\frac{S}{N}\underbrace{I}_{=0})\nonumber \\&-(\mu +\delta )\underbrace{I}_{=0} \nonumber \\= & {} \alpha _1 R +\kappa T\ge 0 \end{aligned}$$
(iii) To verify the $$\frac{\mathrm{{d}}T}{\mathrm{{d}}t}$$ equation satisfies the conditions of Proposition A.1 (Thieme 2018), let $$ T=0$$, and assume $$S,I,R,\ge 0$$. Then
  20 $$\begin{aligned} \frac{\mathrm{{d}}T(t)}{\mathrm{{d}}t}= & {} b(T)(\eta _1 \underbrace{I}_{\ge 0}+\eta _2\frac{R}{N} \underbrace{I}_{\ge 0}+\eta _3\frac{S}{N} \underbrace{I}_{\ge 0}) -(\kappa +\rho +\mu )\underbrace{T}_{=0} \nonumber \\= & {} \frac{\eta _1+\eta _2\frac{ \overbrace{R}^{\ge 0} }{N}+\eta _3\frac{\overbrace{S}^{\ge 0}}{N}}{1+\epsilon \underbrace{T}_{= 0} }\underbrace{I}_{\ge 0} \ge 0 \end{aligned}$$
(iv) To verify the $$\frac{\mathrm{{d}}R}{\mathrm{{d}}t}$$ equation satisfies the conditions of Proposition A.1 (Thieme 2018), let $$ R=0$$, and assume $$I, T,S\ge 0$$. Then
  21 $$\begin{aligned} \frac{\mathrm{{d}}R(t)}{\mathrm{{d}}t}= & {} \omega \underbrace{I}_{\ge 0}+\rho \underbrace{T}_{\ge 0}-\alpha _1\underbrace{R}_{=0}-\alpha _2\underbrace{R}_{=0}\frac{I}{N} -\mu \underbrace{R}_{=0} \nonumber \\= & {} \omega I +\rho T \ge 0 \end{aligned}$$

$$\square $$

Therefore, the coordinate planes and hence the positive octant $\Omega = \{(S,I,T,R)\in {\mathbb {R}}_+^4:0< S,0<I,0< T,0<R\},$ are invariant under the local flow.

#### Theorem 3

All solutions starting in ${\overline{\Omega }} = \{(S,I,T,R)\in {\mathbb {R}}_+^4:0\le S,0\le I,0\le T,0\le R \}$ are bounded forward in time and hence are defined for $[0,\infty )$.

#### Proof

From Eq. (17), we have

$$\begin{aligned} \frac{\mathrm{{d}}N}{\mathrm{{d}}t} =\Lambda -\mu N -\delta I \end{aligned}$$

Since $$N,I>0$$,

$$\begin{aligned} \frac{\mathrm{{d}}N}{\mathrm{{d}}t}\le \Lambda -\mu N. \end{aligned}$$

Therefore,

$$\begin{aligned} N(t)\le \frac{\Lambda }{\mu } +\left( N_0-\frac{\Lambda }{\mu }\right) e^{-\mu t} \end{aligned}$$

where $N_0=N(0)$. Thus, *N* is bounded along solutions for positive times starting in ${\overline{\Omega }}$. Thus, *S*, *I*, *T*, and *R* are also bounded on ${\overline{\Omega }}$ for positive times. Since $\Omega$ is invariant and solutions starting in ${\overline{\Omega }}$ stay bounded for positive times, the solutions exist for all positive times. $\square$

### No Backward Bifurcation for $\epsilon =\alpha _2=0$

In the main text, we (i) numerically demonstrated that a backward bifurcation can exist with $\epsilon =0$ for an unrealistically large $\alpha _2>0$ (120,000 times its estimated baseline value), and (ii) found the analytical curve for the region of bi-stability in $\epsilon$–$\beta$ space showing that a backward bifurcation can exist with realistic $\alpha \ge 0$ for $\epsilon >\epsilon _c(\beta )$. To show that we do not have a backward bifurcation when both $\epsilon =0$ and $\alpha _2=0$, we consider the reduced system of $S'$, $I'$, $T'$ using $R=N-S-I-T$ and letting (1) go to its limiting system $N=(\Lambda -I\delta )/\mu$:

22 $$\begin{aligned} \left\{ \begin{aligned} S'&= \Lambda - \beta S\frac{I\mu }{(\Lambda -I\delta )} -\mu S, \\ I'&= \beta S\frac{I\mu }{(\Lambda -I\delta )}+\alpha _1 \left( \frac{\Lambda -I\delta }{\mu }-S-I-T\right) +\kappa T\\&\quad - \left( \eta _1I+\eta _2I\frac{( (\Lambda -I\delta )/\mu -S-I-T)\mu }{(\Lambda -I\delta )}+\eta _3\frac{IS\mu }{(\Lambda -I\delta )}\right) -(\omega +\mu +\delta )I,\\ T'&=\eta _1I+\eta _2I\frac{( (\Lambda -I\delta )/\mu -S-I-T)\mu }{(\Lambda -I\delta )} +\eta _3\frac{IS\mu }{(\Lambda -I\delta )} -(\kappa +\rho +\mu )T. \end{aligned} \right. \nonumber \\ \end{aligned}$$

We calculate the Jacobian and evaluate it at the DFE:

$$\begin{aligned} {J_{{\mathrm{DFE}}}}=\left[ \begin{matrix} -\mu &{} -\beta &{} 0 \\ -\alpha _1 &{}\displaystyle \frac{-\mu ^2 + (\beta - \eta _1 - \eta _3 - \delta - \omega - \alpha _1)\mu - \alpha _1\delta }{\mu } &{} -\alpha _1+\kappa \\ 0 &{} \eta _1+\eta _3 &{} -(\kappa +\rho +\mu ) \end{matrix}\right] \end{aligned}$$

Following Castillo-Chavez and Song (2004), we take $\beta$ to be the bifurcation parameter and analyze the bifurcation of this system when ${\mathcal {R}}_0=1$ to determine the bifurcation’s direction. We consider ${\mathcal {R}}_0=1$ in (8) and solve for $\beta$:

23 $$\begin{aligned} \beta ^*=\frac{\left( \begin{array}{l}\alpha _1\delta \kappa + \alpha _1\delta \mu + \alpha _1\delta \rho + \alpha _1\eta _1\mu + \alpha _1\eta _3\mu + \alpha _1\kappa \mu + \alpha _1\mu ^2 + \alpha _1\mu \rho + \delta \kappa \mu + \delta \mu ^2 + \delta \mu \rho \nonumber \\ ~\quad + \eta _1\mu ^2 + \eta _1\mu \rho + \eta _3\mu ^2 + \eta _3\mu \rho + \kappa \mu ^2 + \kappa \mu \omega + \mu ^3 + \mu ^2\omega + \mu ^2\rho + \mu \omega \rho \end{array}\right) }{(\kappa + \rho + \mu )(\alpha _1 + \mu )}.\\ \end{aligned}$$

Substituting (23) into the Jacobian at the DFE gives

24 $$\begin{aligned} {J_{{\mathrm{DFE}},\beta =\beta ^*}}=\left[ \begin{matrix} -\mu &{} \quad J_{1,2} &{} \quad 0 \\ -\alpha _1 &{}\quad \displaystyle \frac{\alpha _1}{\mu }J_{1,2} + \frac{(\alpha _1-\kappa )(\eta _1+\eta _2)}{\kappa +\rho +\mu } &{}\quad -\alpha _1+\kappa \\ 0 &{} \quad \eta _1+\eta _3 &{}\quad -(\kappa +\rho +\mu ) \end{matrix}\right] \end{aligned}$$

where

$$\begin{aligned} J_{1,2}= -\frac{[\omega \mu + (\alpha _1 + \mu )(\mu + \delta )](\kappa + \rho + \mu ) + \mu (\alpha _1 + \mu + \rho )(\eta _1 + \eta _3)}{(\kappa + \rho + \mu )(\alpha _1 + \mu )} \end{aligned}$$

We can easily verify that zero is a simple eigenvalue of $J_{{\mathrm{DFE}},\beta =\beta ^*}$ and that all other eigenvalues of $J_{{\mathrm{DFE}},\beta =\beta ^*}$ have negative real part. $J_{{\mathrm{DFE}},\beta =\beta ^*}$ has right eigenvector

$$\begin{aligned} \mathbf{x}=\left[ \begin{matrix} -[\omega \mu + (\alpha _1 + \mu )(\mu + \delta )](\kappa + \rho + \mu ) + \mu (\alpha _1 + \mu + \rho )(\eta _1 + \eta _3) \\ \displaystyle (\kappa + \rho + \mu )\mu (\alpha _1+\mu )\\ \mu (\alpha _1+\mu )(\eta _1 + \eta _3) \end{matrix}\right] \end{aligned}$$

and left eigenvector

$$\begin{aligned} \mathbf{y}=\left[ (\kappa +\rho +\mu ) \alpha _1 \quad -\mu (\kappa +\rho +\mu ) \quad (\alpha _1-\kappa ) \mu \right] . \end{aligned}$$

Writing the right-hand side of our system (22) as *f*, we let $f_k$ be the *k*th component of *f* and set

25 $$\begin{aligned} a= & {} \sum _{k,i,j} y_k x_i x_j \frac{\partial ^2 f_k}{\partial x_i \partial x_j}({\text{ DFE },\beta =\beta ^*}) \nonumber \\ b= & {} \sum _{k,i} y_k x_i \frac{\partial ^2 f_k}{\partial x_i\partial \beta }({\text{ DFE },\beta =\beta ^*}) \end{aligned}$$

For a backward bifurcation to exist, we need $a>0$ and $b>0$. For *b* in (25), its nonzero components are

$$\begin{aligned} \frac{\partial ^2 S'}{\partial S\partial \beta }= & {} \frac{I\mu }{\delta I-\Lambda }, \quad \frac{\partial ^2 S'}{\partial I\partial \beta }= -\frac{\mu S\Lambda }{(\delta I-\Lambda )^2}, \\ \frac{\partial ^2 I'}{\partial S\partial \beta }= & {} -\frac{I\mu }{\delta I-\Lambda }, \quad \frac{\partial ^2 I'}{\partial I\partial \beta }= \frac{\mu S\Lambda }{(\delta I-\Lambda )^2}. \end{aligned}$$

We combine them together using (25), evaluating at the DFE, to get

26 $$\begin{aligned} b=-\frac{\mu (\alpha _1 + \mu )(\kappa + \rho + \mu )\left[ \Lambda S^*\mu ^3 + \Lambda S^*(\alpha _1 + \rho + \kappa )\mu ^2 + \alpha _1\Lambda S^*(\kappa + \rho )\mu \right] }{\Lambda ^2}.\nonumber \\ \end{aligned}$$

Thus, we will always have $b<0$ and a backward bifurcation cannot exist for $\epsilon =\alpha _2=0$

### Coefficients of the $\delta$–$\epsilon$–$\beta$ Equation

The coefficients in Eq. (15) are given in rational form to avoid round off due to decimals:

27 $$\begin{aligned} \nu _6(\delta ,\epsilon ,\beta )= & {} \frac{41\epsilon (136000\delta ^2 + 1940\delta - 1)^2 (\beta - \delta )^2}{32768000000000}, \end{aligned}$$

28 $$\begin{aligned} \nu _5(\delta ,\epsilon ,\beta )= & {} \frac{-1}{32768000000000} (\beta - \delta )(7583360000000000\beta \delta ^3\epsilon \nonumber \\&-\, 7583360000000000\delta ^4\epsilon \nonumber \\&+ \,13940000000\beta \delta ^3 + 162261600000000\beta \delta ^2\epsilon \nonumber \\&- \,13940000000\delta ^4 - 218579200000000\delta ^3\epsilon \nonumber \\&+\, 459274000\beta \delta ^2 + 715778000000\beta \delta \epsilon - 459274000\delta ^3 \nonumber \\&-\, 1491252000000\delta ^2\epsilon - 268440\beta \delta \nonumber \\&-\, 397700000\beta \epsilon + 268440\delta ^2 + 1209500000\delta \epsilon \nonumber \\&+ \,33\beta - 33\delta - 205000\epsilon ), \end{aligned}$$

29 $$\begin{aligned} \nu _4(\delta ,\epsilon ,\beta )= & {} \frac{-141921}{655360}\beta \delta ^2 + \frac{17425}{4096}\delta ^4 - \frac{6711}{327680000}\beta ^2 \nonumber \\&- \,\frac{4351}{65536000}\delta ^2 + \frac{199}{32768000000}(\delta -\beta ) \nonumber \\&+ \,\frac{41}{5242880}\epsilon + \frac{111084375}{128}\delta ^4\epsilon + \frac{52275}{16384}\beta ^2\delta ^2 \nonumber \\&-\, \frac{121975}{16384}\beta \delta ^3 + \frac{25876125}{1024}\delta ^3\epsilon + \frac{229637}{3276800}\beta ^2\delta \nonumber \\&+ \,\frac{1789445}{65536}\beta ^2\epsilon + \frac{5817695}{32768}\delta ^2\epsilon + \frac{14233}{163840000}\beta \delta \nonumber \\&+ \,\frac{4059}{65536}\beta \epsilon - \frac{12259}{131072}\delta \epsilon + \frac{14999}{102400}\delta ^3 \nonumber \\&+ \,\frac{25353375}{2048}\beta ^2\delta \epsilon - \frac{77105625}{2048}\beta \delta ^2\epsilon \nonumber \\&-\, \frac{5666815}{32768}\beta \delta \epsilon + \frac{111084375}{128}(\beta ^2\delta ^2\epsilon -2\beta \delta ^3\epsilon ),\end{aligned}$$

30 $$\begin{aligned} \nu _3(\delta ,\epsilon ,\beta )= & {} \frac{-32671875}{4096}(2\delta ^3 + \beta ^2\delta ) - \frac{5740925}{65536}\beta ^2 \nonumber \\&-\, \frac{18774825}{32768}\delta ^2 - \frac{3761}{32768}\beta + \frac{23377}{131072}\delta \nonumber \\&+\, \frac{2588125}{32768}\epsilon + \frac{23142578125}{16}(2\beta \delta ^2\epsilon - \beta ^2\delta \epsilon -\delta ^3\epsilon )\nonumber \\&- \,\frac{5281953125}{512}\beta ^2\epsilon + \frac{98015625}{4096}\beta \delta ^2 \nonumber \\&-\, \frac{1361328125}{32}\delta ^2\epsilon + \frac{18257475}{32768}\beta \delta + \frac{1211678125}{8192}\beta \epsilon \nonumber \\&-\,\frac{1259340625}{4096}\delta \epsilon - \frac{233}{26214400} \nonumber \\&+ \,\frac{27063203125}{512}\beta \delta \epsilon , \end{aligned}$$

31 $$\begin{aligned} \nu _2(\delta ,\epsilon ,\beta )= & {} \frac{-3911421875}{8192}\beta + \frac{6806640625}{1024}(\beta ^2 + 4\delta ^2) \nonumber \\&+ \,\frac{14464111328125}{16}(\beta ^2\epsilon + \delta ^2\epsilon -2\beta \delta \epsilon ) \nonumber \\&- \,\frac{34033203125}{1024}\beta \delta + \frac{3437353515625}{128}(\delta \epsilon -\beta \epsilon ) \nonumber \\&- \,\frac{5208125}{32768} + \frac{2036546875}{2048}\delta , \end{aligned}$$

32 $$\begin{aligned} \nu _1(\delta ,\epsilon ,\beta )= & {} \frac{-2646728515625}{4096} + \frac{4254150390625}{256}(\beta -\delta ). \end{aligned}$$

### Additional PRCC Plots and Discussion

Here we find the tables and figures of other variables that may be of interest. For the variable of Yearly completed treatment, see Fig. 10 and Table 4. The Yearly completed treatment variable keeps track of the number of individuals who were in treatment and have moved into the recovered class. For the variable of Yearly treatment, see Fig. 11 and Table 4. The Yearly treatment variable keeps track of the number of individuals who went to treatment. For the variable of Yearly *I* to *R*, see Fig. 12 and Table 4. The Yearly *I* to *R* variable keeps track of the number of individuals who left the HUD class by either quitting on their own or with the help of a non-specialty treatment facility. For the variable *T*, see Fig. 13 and Table 5. The *T* variable keeps track of the number of individuals in the *T* class. For the variables *S*, *I*, and *R*, see Figs. 14,  15, and Table 5. The *S* variable keeps track of the number of individuals in the HUD class. The *I* variable keeps track of the number of individuals in the *I* class. The *R* variable keeps track of the number of individuals in the *R* class.

**Table 4:** PRCC results for those that completed treatment (yearly completed treatment), those that went to treatment (yearly treatment), and those who are in those who left the HUD class either quitting on their own or with the help of a non-specialty treatment facility (yearly *I* to *R*), using baseline parameters (5) and either constant $$\delta =0.03002$$ or the variable $$\delta $$ in (16). The initial conditions for $$t=0$$ in 2020 were generated using (4)–(5), 2002 values of $$S= 199{,}500$$, $$I=102$$, $$T= 95 $$, $$R= 100$$, and running the system until 2020 (as previously described to obtain Fig. 2). The PRCC values at 2030 are given here with the columns labeled “constant” corresponding to the constant death rate of $$\delta = 0.03002$$ (its extrapolated 2020 value) and the columns “variable” corresponding to the variable death rate defined in (16)

IC/param	Yearly completed treatment		Yearly treatment		Yearly *I* to *R*	
IC/ param	Constant	Variable	Constant	Variable	Constant	Variable
*S*(0)	–	–	–	–	–	–
*I*(0)	0.91	0.87	0.89	0.90	0.92	0.94
*T*(0)	0.65	0.61	0.58	0.65	0.68	0.73
*R*(0)	0.51	0.41	0.42	0.51	0.55	0.59
$\Lambda \ *$	–	–	–	–	–	–
$\mu$	$-$ 0.40	–	–	–	–	$-$ 0.48
$\beta$	0.88	0.85	0.89	0.91	0.90	0.93
$\eta _1$	0.84	0.78	0.82	0.82	$-$ 0.52	$-$ 0.63
$\eta _2 \ *$	–	–	–	–	–	–
$\eta _3$	0.44	0.48	0.45	0.48	–	$-$
$\rho$	0.98	0.97	–	–	–	–
$\kappa$	$-$ 0.84	$-$ 0.85	0.90	0.92	0.57	0.63
$\alpha _1$	0.59	0.52	0.52	0.59	0.58	0.66
$\alpha _2 \ *$	–	–	–	–	–	–
$\delta$	$-$ 0.49	–	$-$ 0.55	–	$-$ 0.63	–
*m*	–	–	–	–	–	–
*b*	–	$-$ 0.40	–	$-$ 0.59	–	$-$ 0.65
$\omega$	–	–	–	–	0.94	0.96
$\epsilon \ *$	$-$ 0.85	$-$ 0.82	$-$ 0.80	$-$ 0.83	0.54	0.55

All table entries without a value are not significant. The notation of * denotes that the parameter does not appear in the formula for ${\mathcal {R}}_0$. The corresponding graphs for this table are given in Figs. 10, 11, and 12

**Table 5:** PRCC results for those that are susceptible (model output *S*), those that are in the HUD class (model output *I*), those who are in treatment (model output *T*), and those that have recovered (model output *R*), using baseline parameters (5) and either constant $$\delta =0.03002$$ or the variable $$\delta $$ in (16). The initial conditions for $$t=0$$ in 2020 were generated using (4)–(5), 2002 values of $$S= 199{,}500$$, $$I=102$$, $$T= 95 $$, $$R= 100$$, and running the system until 2020 (as previously described to obtain Fig. 2). The PRCC values at 2030 are given here with the columns labeled “constant” corresponding to the constant death rate of $$\delta = 0.03002$$ (its extrapolated 2020 value) and the columns “variable” corresponding to the variable death rate defined in (16)

IC/ param	*S*		*I*		*T*		*R*	
	Constant	Variable	Constant	Variable	Constant	Variable	Constant	Variable
*S*(0)	0.99	0.99	–	–	–	–	–	–
*I*(0)	–	–	0.94	0.98	0.93	0.89	0.91	0.94
*T*(0)	–	–	0.72	0.85	0.72	0.63	0.63	0.73
*R*(0)	–	–	0.58	0.76	0.55	–	0.68	0.74
$\Lambda \ *$	0.93	0.91	–	–	–	–	–	–
$\mu$	$-$ 0.93	$-$ 0.91	$-$ 0.40	$-$ 0.63	$-$ 0.41	–	–	$-$ 0.52
$\beta$	–	–	0.93	0.97	0.90	0.84	0.81	0.84
$\eta _1$	–	–	$-$ 0.56	$-$ 0.80	0.87	0.78	–	–
$\eta _2 \ *$	–	–	–	–	–	–	–	–
$\eta _3$	–	–	$-$ 0.23	$-$ 0.35	0.49	0.44	–	–
$\rho$	–	–	–	$-$ 0.42	$-$ 0.66	$-$ 0.55	0.81	0.86
$\kappa$	–	–	0.58	0.78	$-$ 0.87	$-$ 0.81	–	–
$\alpha _1$	–	–	0.66	0.82	0.58	0.48	$-$ 0.94	$-$ 0.96
$\alpha _2 \ *$	–	–	–	–	–	–	–	–
$\delta$	–	–	$-$ 0.66	–	$-$ 0.56	–	–	–
*m*	–	–	–	0.49	–	–	–	–
*b*	–	–	–	$-$ 0.81	–	$-$ 0.50	–	$-$ 0.44
$\omega$	–	–	$-$ 0.46	$-$ 0.61	–	–	0.83	0.89
$\epsilon \ *$	–	–	0.61	0.75	$-$ 0.87	$-$ 0.79	–	–

All table entries without a value are not significant. The notation of * denotes that the parameter does not appear in the formula for ${\mathcal {R}}_0$. The corresponding graphs for this table are given in Figs. 14, 13, and 15
